# Heterologous Gene Regulation in Clostridia: Rationally Designed Gene Regulation for Industrial and Medical Applications

**DOI:** 10.1021/acssynbio.2c00401

**Published:** 2022-10-20

**Authors:** Yanchao Zhang, Tom S. Bailey, Aleksandra M. Kubiak, Philippe Lambin, Jan Theys

**Affiliations:** † The M-Lab, Department of Precision Medicine, GROW - School of Oncology and Reproduction, Maastricht University, 6229 ER Maastricht, The Netherlands; ‡ Exomnis Biotech BV, Oxfordlaan 55, 6229 EV Maastricht, The Netherlands

**Keywords:** glucuronidase, nitroreductase, promoter-5′ untranslated region, alternative start codon, *sacB* gene, clostridia

## Abstract

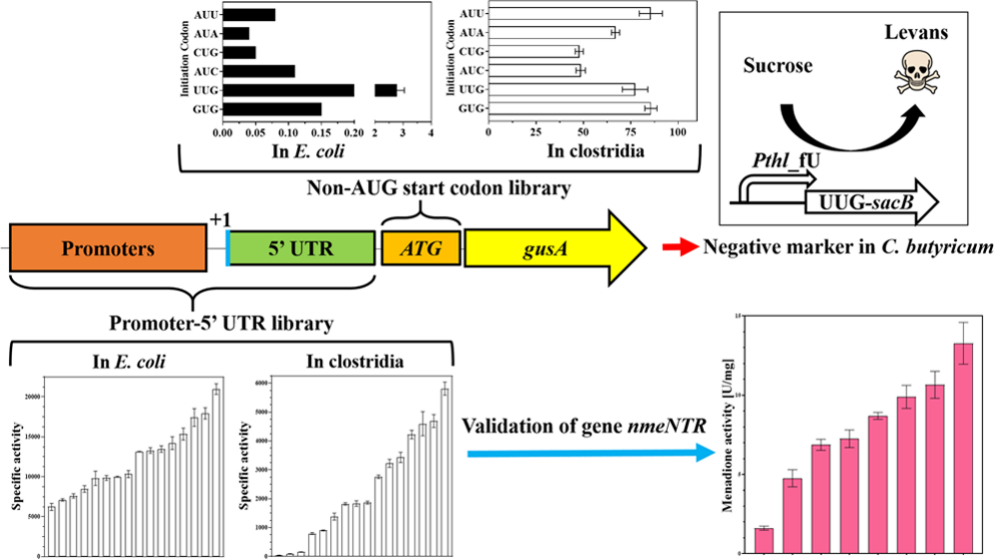

Several species from the *Clostridium* genus show promise as industrial solvent producers and cancer therapeutic delivery vehicles. Previous development of shuttle plasmids and genome editing tools has aided the study of these species and enabled their exploitation in industrial and medical applications. Nevertheless, the precise control of gene expression is still hindered by the limited range of characterized promoters. To address this, libraries of promoters (native and synthetic), 5′ UTRs, and alternative start codons were constructed. These constructs were tested in *Escherichia coli* K-12, *Clostridium sporogenes* NCIMB 10696, and *Clostridium butyricum* DSM 10702, using β-glucuronidase (*gusA*) as a gene reporter. Promoter activity was corroborated using a second gene reporter, nitroreductase (*nmeNTR*) from *Neisseria meningitides*. A strong correlation was observed between the two reporters. In *C. sporogenes* and *C. butyricum*, respectively, changes in GusA activity between the weakest and strongest expressing levels were 129-fold and 78-fold. Similar results were obtained with the *nmeNTR*. Using the GusA reporter, translation initiation from six alternative (non-AUG) start codons was measured in *E. coli*, *C. sporogenes*, and *C. butyricum*. Clearly, species-specific differences between clostridia and *E. coli* in translation initiation were observed, and the performance of the start codons was influenced by the upstream 5′ UTR sequence. These results highlight a new opportunity for gene control in recombinant clostridia. To demonstrate the value of these results, expression of the *sacB* gene from *Bacillus subtilis* was optimized for use as a novel negative selection marker in *C. butyricum*. In summary, these results indicate improvements in the understanding of heterologous gene regulation in *Clostridium* species and *E. coli* cloning strains. This new knowledge can be utilized for rationally designed gene regulation in *Clostridium*-mediated industrial and medical applications, as well as fundamental research into the biology of *Clostridium* species.

## Introduction

*Clostridium* is a highly diverse genus of Gram-positive bacteria, unified by their ability to form endospores and their inability to grow in the presence of oxygen. Although several hundred species are taxonomically classified as clostridia, more recent phylogenetic analysis suggests that a much smaller group of bacteria are evolutionarily related, the so-called “*Clostridium sensu stricto*”.^1^ Historically, species of this genus were synonymous with infection, caused by species such as *Clostridium botulinum*, *Clostridium tetani*, *Clostridium perfringens*, and *Clostridioides difficile*. However, most clostridia are completely benign.^2^ Beyond the domain of human health, solventogenic clostridia have been utilized in the industrial production of petrochemicals due to their ability to produce acetone–butanol–ethanol (ABE) by microbial fermentation (*Clostridium acetobutylicum*, *Clostridium beijerinckii*, *Clostridium saccharobutylicum*, and *Clostridium saccharoperbutylacetonicum*),^3^ while cellulolytic, biofuel-producing strains, such as *Clostridium cellulolyticum* and *Clostridium cellulovorans*, are now attracting attention to satisfy the increasing demand for cleaner fuels.^4,5^ Oncolytic strains, such as *Clostridium sporogenes* and *Clostridium novyi*, selectively germinate in the hypoxic/necrotic regions of tumors and their endogenous oncolytic activity can result in partial destruction of the tumor.^6,7^ Subsequent studies have highlighted distinct advantages to using certain *Clostridium* species as a therapeutic vector in bacterial-mediated cancer therapy.^8−10^ The addition, through genetic engineering, of clinically approved heterologous products, such as antibodies,^11^ proinflammatory cytokines,^12,13^ and checkpoint inhibitors, could further increase their therapeutic value. An alternative approach is clostridia-directed enzyme prodrug therapy (CDEPT), which has seen significant advances in recent years.^14−18^ Thus, demand for synthetic strains of these species has highlighted the need for genetic tools that will enable the precise control of synthetic products of industrial or therapeutic value. This need has been met to a degree in the form of an *Escherichia coli*–*Clostridium* shuttle plasmids,^2^ the bacterial group II intron technology (ClosTron),^19^ and CRISPR-based editing systems.^20,21^

Accurate quantification of available gene promoters enables synthetic biologists to control recombinant bacteria precisely. In the industrial context, this enables microorganisms to be modified for maximum product yield and minimum cellular biomass.^22^ Intratumoral delivery of therapeutics will have similar demands. However, promoters reported in the literature are typically of *Clostridium* origin and from a very limited number of species, such as the promoters of the *thl* (thiolase), *ptb* (phosphotransbutyrylase), and *araE* (arabinose sugar–proton symporter) genes of *C. acetobutylicum* ATCC 824^23−25^ and of the *fdx* (ferredoxin) gene of *C. sporogenes* NCIMB 10696.^26^ Due to the limited range of strengths and the multitude of regulatory proteins that can interfere with native promoters, the focus of researchers has shifted to the generation of synthetic promoter libraries.^27^ In a recent study, the widely used constitutive promoter of *C. acetobutylicum*, P*thl*, was employed to generate synthetic promoters by randomization of the regions surrounding the consensus −35 and −10 elements. The apparent stringent requirements for promoters in *Clostridium* reduce the number of functional promoters in synthetic libraries, highlighting the challenge of controlling gene expression in this genus.^28,29^ In addition to controlling transcript production by the promoter, the 5′ untranslated region (5′ UTR) of the resultant mRNA, essential for translation, is another element that can be exploited for controlling gene expression.^30^ Moreover, the start codon is recognized by the ribosome to initiate translation^31^ and is a key modulator of translation.^32^

Here, we describe the generation of promoters, 5′ UTRs, and alternative start codon libraries that enable genes to be expressed at different levels in clostridia. The promoter-5′ UTR library builds on previously published work, while the alternative start codon library is novel and adds to the repertoire of elements for gene control in clostridia. First, we optimized the construction of a glucuronidase (GusA) reporter system for the rapid generation expression variants. Using this system, we directly compared frequently cited native promoters of *Clostridium* in an established GusA assay, which showed good stability and sensitivity. Second, consensus promoter sequences, based on whole genome promoter alignment, were predicted. In combination with different 5′ UTR sequences, this promoter-5′ UTR library was evaluated for gene expression in two *Clostridium sensu stricto* species: *C. sporogenes*-NT^12^ and *C. butyricum* DSM 10702.^33^ This library was cross-validated using a previously reported nitroreductase of *Neisseria meningitidis*, NmeNTR^14^ in *C. sporogenes*-NT. In addition, we measured translation initiation from alternative (non-AUG) start codons in *E. coli*, *C. sporogenes*-NT, and *C. butyricum* by the GusA assay. Finally, to demonstrate the value of these results, we tuned the expression of the *sacB* gene, encoding levansucrase, from *Bacillus subtilis* and reported its use as a novel negative selection maker in *C. butyricum*.

## Results and Discussion

### Design and Golden Gate Construction of the GusA Reporter System in *E. coli* and Clostridia

A range of gene products has been utilized as gene expression reporters. To serve this purpose, the protein should be stable, sensitive enough to detect the signal of weak promoters above background noise, inexpensive, and simple to assay. Fluorescent proteins, such as GFP or RFP, are appealing due to the simplicity of signal detection, but the oxygen requirement of these reporters limits their use in anoxic conditions. Recently, oxygen-independent fluorescent reporters have been developed in clostridia, including iLOV,^34^ FAST,^35^ HaloTag, and SNAP-tag proteins.^36^ Fluorescent reporters are nonenzymatic and nonamplifying, which could reduce their sensitivity for quantifying gene expression compared to enzyme-based reporter assays.^28,37^ Similarly, chloramphenicol acetyl transferase (CAT) of the antibiotic selection marker is a popular reporter in clostridia^38−40^ that has been used previously to generate a synthetic promoter library.^29^ Due to the lack of sensitivity, relatively weak promoters may fall below the detection limit of CAT, limiting its use as a reporter.^41^ Enzymatic reporters have been widely used in clostridia, including β-galactosidase (LacZ)^23,42−44^ and glucuronidase (GusA). Since the GUS reporter system of plants was reviewed in 1989, most researchers in clostridia have employed the GusA reporter to evaluate promoter strength.^28,45−48^ Based on these considerations, we here chose GusA as a sensitive and reliable reporter that allows the comparison of strong and weak promoters covering a broad dynamic range of gene expression.

**Figure 1:**
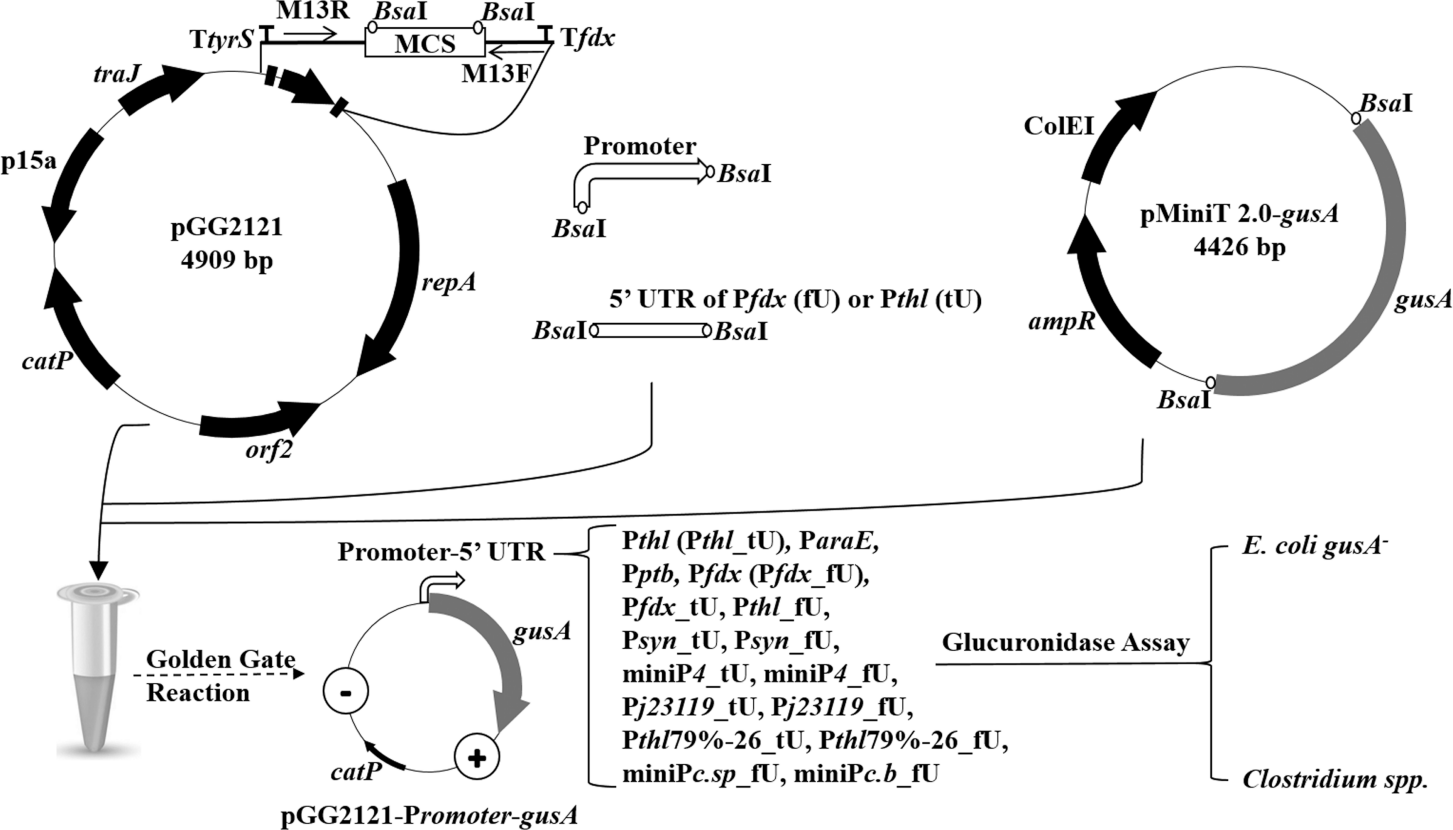
Schema of the GusA reporter system. *catP*, thiamphenicol or chloramphenicol resistance gene; *ampR*, ampicillin resistance gene; p15a and ColE1 (−), Gram- replicons; *orf2* and *repA* (+), Gram+ replicon; *traJ*, conjugal transfer gene; T*tyrS* and T*fdx*, terminators; MCS, multiple cloning site; M13R and M13-F, universal primer pairs M13-F/R.

To streamline the process of cloning multiple promoters and 5′ UTRs, a cloning strategy based on the golden gate technique was developed. To facilitate the generation of plasmid-based promoter-5′ UTR-GusA constructs at minimal time and monetary cost, a vector with two *Bsa*I restriction sites in the multiple cloning site was generated. The first version of this vector (pGG2151) was based on the high-copy *E. coli*–*Clostridium* shuttle vector, pMTL82151. Initial attempts to use this vector in Golden Gate assembly reactions yielded low-efficiency assembly with a high rate of single nucleotide polymorphisms (SNPs). Sanger sequencing showed that the SNPs frequently occurred in the ribosome-binding site (RBS) and the open reading frame (ORF) of *gusA* gene. We speculated that due to the high-copy number of the Gram-negative replicon ColE1 in *E. coli*,^49^ the resulting high expression level of the *gusA* gene was harmful to the host and resulted in cloning difficulties. Thus, the replicon was swapped to low-copy p15a replicon (pGG2121; Figure 1). Subsequent assemblies showed very high efficiency, and the occurrence of SNPs was rare, suggesting that the low-copy number is more stable for cloning the GusA reporter system compared to the high-copy number.

Figure 1 shows a schematic illustration of the vector design and cloning workflow. GusA expression plasmids (pGG2121-P*romoter*-*gusA*) were assembled utilizing the Golden Gate reaction and transformed into *E. coli* K-12 strain JW1609 (*gusA*^*–*^)^50^ or *E. coli* S17-1 for conjugation into *Clostridium* spp. *Clostridium* transconjugants were confirmed by Sanger sequencing before being tested in the GusA assay. This workflow enabled the library to be generated faster and more efficiently, as compared to conventional cloning techniques.

### Effective GusA Reporter Evaluates Promoter Strengths

**Figure 2:**
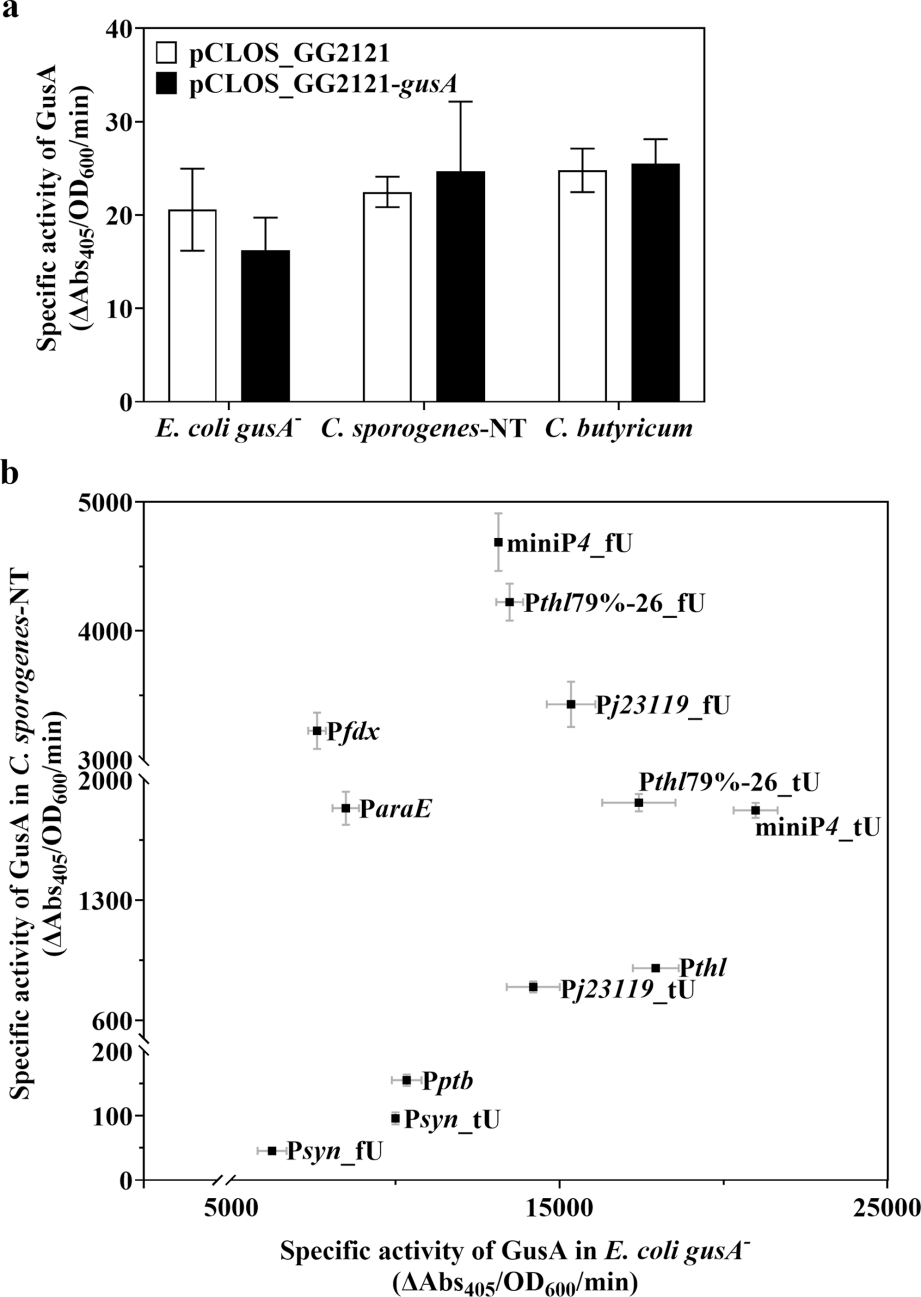
GusA reporter available to evaluate different promoters in *E. coli* and *Clostridium*. (a) Specific activities of glucuronidase of negative controls pGG2121 and pGG2121-*gusA* in *E. coligusA*^*–*^, *C. sporogenes*-NT, and *C. butyricum*. (b) Strengths of native promoters (P*thl*, P*fdx*, P*ptb*, P*araE*), promoter_tU, and promoter_fU fusions in *E. coligusA*^–^ and *C. sporogenes*-NT by glucuronidase assay. Data represent the mean ± s.d. of three biological replicates.

To enable comparison of expression levels, all samples were obtained at the same growth point (OD_600_ = 1) across all constructs for both *E. coli* and *Clostridium* strains harboring GusA expression plasmids. As controls, strains carrying an empty vector pGG2121 or a promoter-less *gusA* vector (pGG2121-*gusA*) were assayed. Both constructs demonstrated very low GusA activity in *E. coligusA*^*–*^, *C. sporogenes*-NT, and *C. butyricum* (Figure 2a). These observations confirmed the absence of endogenous glucuronidase activity in these strains and showed that the *gusA* expression cassette is insulated from the effects of other promoters from the vector pGG2121. Next, we evaluated four native promoters from *Clostridium* spp., which have previously been characterized and widely used for gene expression, including P*thl*,^23,44,51−54^ P*fdx*,^2,14,15,55^ P*ptb,*^23,35,44,52^ and P*araE*.^23,26,56^ As shown in Figure 2b, among these four native promoters, P*fdx* showed the highest strength in *C. sporogenes*-NT but was among the weakest in *E. coli*. The GusA activity levels obtained for P*araE*, P*thl*, and P*ptb* in *C. sporogenes*-NT correspond to previous results recorded in *C. ljungdahlii*.^23^ In their study, P*thl* and P*araE* promoters exhibited much greater LacZ activity at all three time points than P*ptb* and P*adc* promoters (encoding the acetoacetate decarboxylase gene in *C. acetobutylicum* ATCC 824).

All four native *Clostridium* promoters showed significant GusA activity in *E. coli* (Figure 2b). Similarly, almost all of the synthetic promoters generated by conventional randomization were active in *E. coli* but not in clostridia.^28^ These results suggest that sigma factors of *E. coli* permit to function on promoter sequences from distantly related species. Overall, the data demonstrate that the GusA reporter system serves as a useful tool for the validation of promoter strengths and their comparison across species.

### Promoters with Altered 5′ UTR Exhibit Various Strengths

We initially tested four native *Clostridium* promoters in our reporter system. Yet, screening for native promoters is time-consuming.^21^ Thus, in many species, researchers apply the approach of randomizing specific regions of the promoter surrounding essential, conserved regions to rapidly generate synthetic promoter libraries.^27,57^ In *Clostridium spp*., the generation of synthetic promoter libraries by sequence randomization of P*thl* has been published by Mordaka et al.^28^ and Yang et al.^29^ Despite the conservation of consensus promoter sequences, the randomization of flanking sequences generated numerous promoters that remained active in *E. coli* but were completely inactive in clostridia. These studies highlighted the stringent requirements for functional promoters in clostridia, which could prove a significant obstacle to gene overexpression in these species. Bioinformatics tools offer an alternative approach to mutagenesis methods for finding novel gene promoters. A method involves aligning large numbers of promoter motifs, extracted from the genome sequence, to determine the most common promoter. This approach has been applied with success for the expression of guide RNAs in CRISPR systems in *Clostridium* spp., such as miniP*4* in *C. cellulolyticum*,^58^ P*syn* in *C. sporogenes* ATCC 15579,^59^ and P*j23119* in multiple *Clostridium* species.^26,60,61^ The lack of a 5′ UTR in these constructs, containing the RBS that is essential for translation, does not enable a protein-level assessment of gene expression, as is required for the GusA reporter. To include these promoters (miniP*4*, P*syn*, and P*j23119*) in our study, we added the native 5′ UTR of P*thl* (tU) to the 3′ end of the promoters, resulting in the promoter-5′ UTR fusions miniP*4*_tU, P*syn*_tU and P*j23119*_tU. As shown in Figure 2b, all three promoter-5′ UTR sequences were active in *C. sporogenes*-NT. miniP*4*_tU showed a similar level of expression to P*thl*79%-26_tU, which was the strongest promoter in a previous P*thl* mutant library.^28^ The results suggest that promoter prediction by bioinformatics tools could be an efficient method for finding functional promoters of different strengths in the *Clostridium* genus.

**Figure 3:**
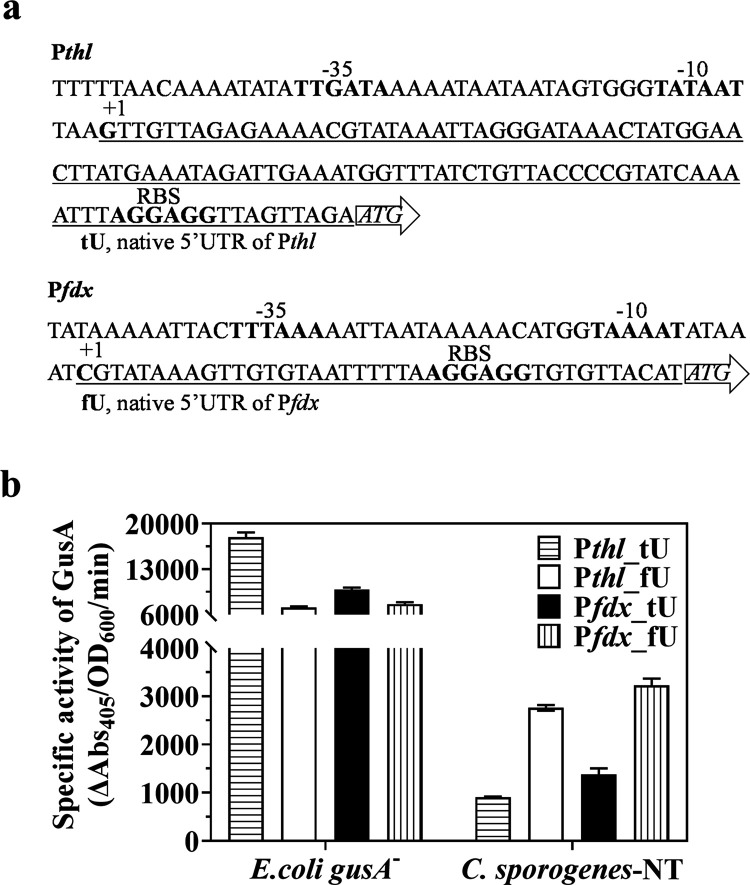
Evaluation of promoters with altered 5′ UTR. (a) The native 5′ UTR of P*thl* and P*fdx* is underlined. The −10 region, −35 region, ribosome-binding site (RBS), and transcription start site (TSS) are in bold and annotated. The arrow with ATG represents the gene of interest. (b) Strengths of P*fdx*_tU, P*fdx*_fU, P*thl*_tU, and P*thl*_fU in *E. coligusA*^–^ and *C. sporogenes*-NT by glucuronidase assay. Data represent the mean ± s.d. of three biological replicates.

As shown in Figure 2b, native promoter P*fdx* exhibited the highest GusA activity in our current library (specific activity of 3000). Both processes of transcription and translation affect gene expression level. The 5′ UTR plays a critical role in a fine balance between transcription, transcript stability, and translation.^30^ To investigate the influence of the 5′ UTR on gene expression, we directly exchanged tU with the 5′ UTR of P*fdx* (fU), which is considerably shorter than tU (Figure 3a), resulting in promoter-5′ UTR fusions P*thl*79%-26_fU, miniP*4*_fU, P*syn*_fU, and P*j23119*_fU. In *C. sporogenes*-NT, P*thl*79%-26_fU, miniP*4*_fU, and P*j23119*_fU showed approximately 2-fold, 2.5-fold, and 4-fold higher GusA activities than those with tU, respectively. Of all of the promoters-5′ UTR tested, P*syn*_fU and P*syn*_tU showed the lowest GusA activity in both *C. sporogenes*-NT and *E. coli* (Figure 2b), possibly due to the weak strength of the P*syn* promoter itself. In Figure 2b, the replacement of tU with fU weakened GusA expression in *E. coli*. We hypothesize that the fU negatively affects gene expression in *E. coli*, while increasing the expression in *Clostridium*. Thus, we applied the 5′ UTR exchange to the native promoters P*thl* (P*thl*_tU) and P*fdx* (P*fdx*_fU), obtaining modified promoter-5′ UTR fusions P*thl*_fU and P*fdx*_tU. In *C. sporogenes*-NT, the GusA activity of P*thl*_fU was higher than that of the P*thl*_tU native promoter, while P*fdx*_tU showed a significant decrease in GusA activity compared to native P*fdx*_fU. The reverse was observed in *E. coli* (Figure 3b).

Regulatory events acting on the 5′ UTR of gene transcripts may differ significantly between *E. coli* and *Clostridium* spp. The full complement of 5′ UTR regulation is not well understood, even in the model bacterium *E. coli*. The different lengths of fU and tU of the 5′ UTRs might affect mRNA stability involved in the degradation of RNA,^62^ as has been observed when short stem loops are added to the 5′ UTR to enhance gene expression in *C. acetobutylicum*.^63^ The 5′ UTR of P*fdx* was investigated in a *Clostridium* riboswitch study. Addition of fU improved transcript abundance in clostridia,^26^ which could be a plausible explanation for our findings. Another explanation is that translation initiation rates between fU and tU could be different, possibly due to the Shine–Dalgarno sequence.^64^ Our results indicate that the use of the 5′ UTR of P*fdx* augments gene expression in clostridia via an unknown mechanism. This could serve as a template for future *Clostridium* 5′ UTR studies. Our results demonstrate that altering 5′ UTR downstream of promoters could significantly regulate gene expression levels. Overall, this modification enabled us to expand the library with 10 additional promoters with altered 5′ UTR, of which miniP*4*_fU showed the highest GusA activity in *C. sporogenes* (specific activity of 4700; Figure 2b).

### Applicable GusA Promoter-5′ UTR Library in Clostridia

**Figure 4:**
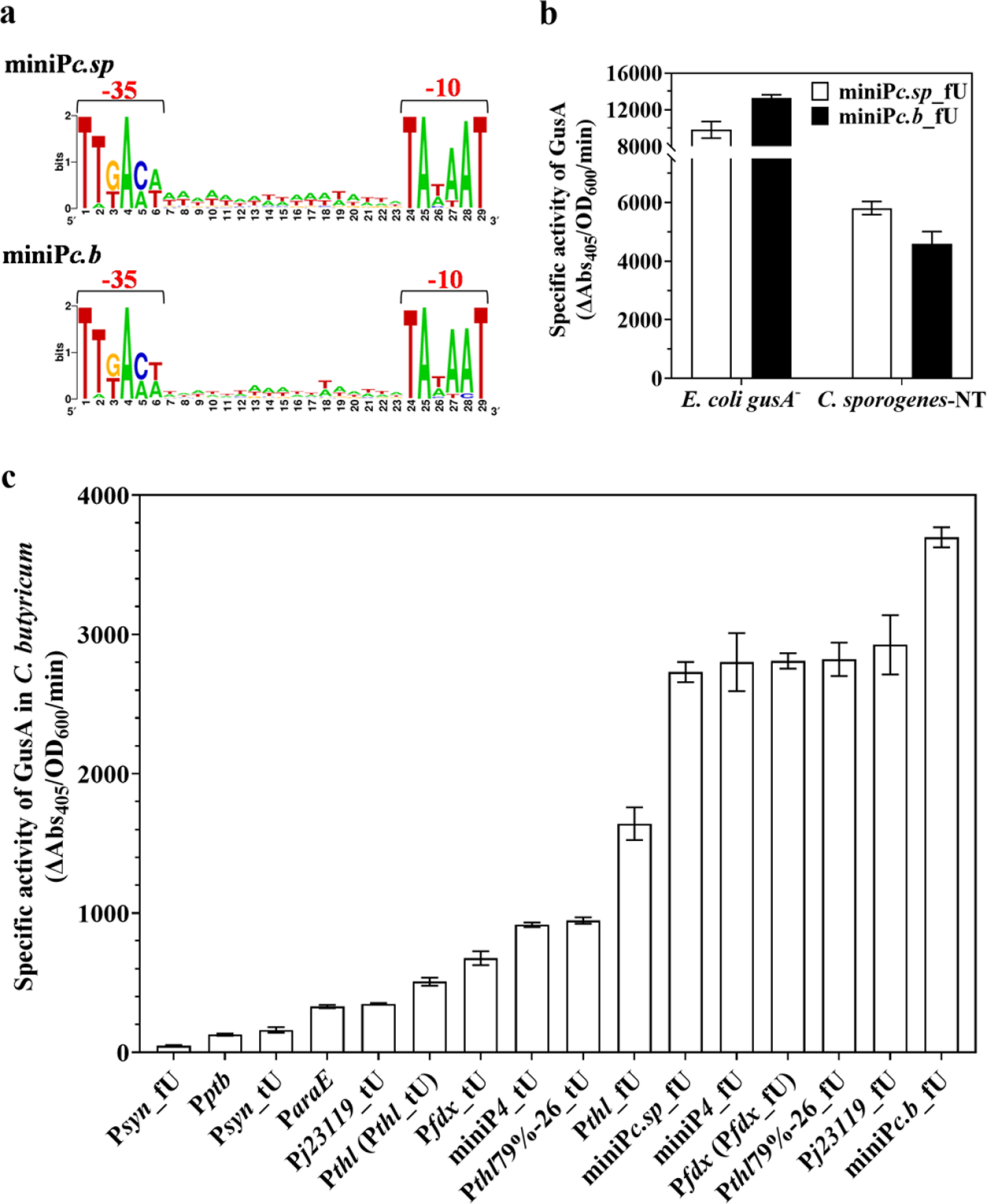
Applicability of the GusA promoter-5′ UTR library. (a) Alignments of predicting promoter from *C. sporogenes* NCIMB 10696 (miniP*c.sp*) and *C. butyricum* DSM 10702 (miniP*c.b*). A 17-nt AT-rich spacer separates two highly conserved regions (−35 and −10 in red highlight). (b) Strengths of newly constructed promoters-5′ UTR miniP*c.sp*_fU and miniP*c.b*_fU in *E. coligusA-* and *C. sporogenes*-NT by glucuronidase assay. (c) Distribution of promoter-5′ UTR library in *C. butyricum* by glucuronidase assay. Data represent the mean ± s.d. of three biological replicates.

A native “consensus” promoter can be determined by the alignment of predicted native promoters using the webserver PePPER.^65^ This produced good results in *C. cellulolyticum* H10 (NC_011898.1).^58^ The resulting promoter miniP*4* produced a very high level of gene expression in our reporter assay, suggesting that this method can generate strong promoter candidates that perform well between related species. Thus, to create novel predicted consensus promoters using the same method, we used the genomes of *C. sporogenes* NCIMB 10696 (NZ_CP009225.1) and *C. butyricum* DSM 10702 (NZ_CP040626.1) as input data for the PePPER algorithm. For each species, 363 and 142 promoters, respectively, were identified (Table S3). These were aligned to create two 29-nucleotide-long DNA logos using WebLogo,^66^ named miniP*c.sp* and miniP*c.b*, respectively (Figure 4a). The logos determine the conserved −10 and −35 sequences and the variable, AT-rich, intermediate sequences. The most common sequence was determined for each species, and this sequence was cloned with fU into the GusA reporter system. As shown in Figure 4b, both constructs produced high GusA activity in *C. sporogenes*-NT and *E. coli*. MiniP*c.sp*_fU exhibited the highest GusA activity in the promoter-5′ UTR library of *C. sporogenes*-NT (Figure S1), over 1.8-fold higher than that of native promoter P*fdx*. The 16 promoters-5′ UTR of the library in *C. sporogenes* showed a broad range of GusA expression, with a 129-fold change between the weakest (P*syn*_fU) and the strongest (miniP*c.sp*_fU) promoters-5′ UTR (Figure S1).

To demonstrate the portability and utility of this library in the *Clostridium* genus, all 16 promoters-5′ UTR expressing GusA were transferred into the distantly related *Clostridium* species *C. butyricum* DSM 10702. This species is a butyric acid-producing strain of industrial relevance^67^ and is widely used as a probiotic.^68,69^ All 16 promoters-5′ UTR were active and exhibited a wide range of strengths (over 78-fold change; Figure 4c). As shown in Figures S1 and 4c, the GusA promoter-5′ UTR library showed a good correlation between *C. sporogenes* and *C. butyricum*, suggesting that this collection of promoters-5′ UTR could be generally applied in clostridia. In addition, miniP*c.b*_fU exhibited the highest GusA activity in *C. butyricum* (Figure 4c), suggesting that strong promoters can be designed for one specific clostridia using the method employed here. Overall, a collection of promoter-5′ UTR sequences (Table S2), functional in two divergent *Clostridium* species, was generated using native promoters, bioinformatics promoter prediction, and native 5′ UTR exchange.

### Expressing Nitroreductase by Promoter-5′ UTR Library in *C. sporogenes*

**Figure 5:**
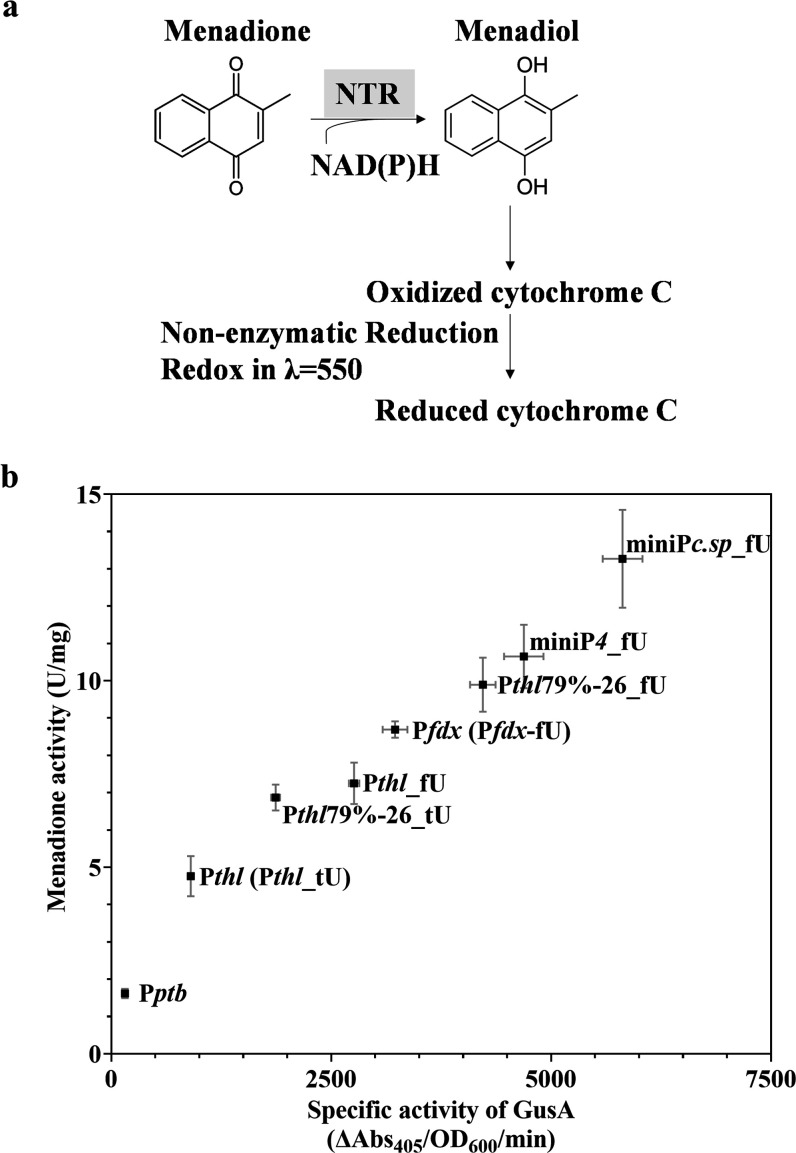
Expressing *nmeNTR* to validate the promoter-5′ UTR library in *C. sporogenes*. (a) The scheme of menadione nitroreductase assay. (b) The menadione nitroreductase and glucuronidase activities of selected promoters-5′ UTR in *C. sporogenes*-NT. Data represent the mean ± s.d. of three biological replicates.

To validate the results of activity levels obtained from the GusA assay and to show its potential usefulness in *Clostridium*-mediated medical applications, eight promoters (P*ptb*, P*thl*, P*fdx*, P*thl*_fU, P*thl*79%-26_tU, miniP*4*-fU, P*thl*79%-26_fU, and miniP*c.sp*_fU) were selected from the library to express the previously characterized nitroreductase gene from *Neisseria meningitidis*, *nmeNTR*, as a therapeutic gene. NmeNTR was studied as a prodrug converting enzyme (PCE) in *Clostridium*-directed enzyme prodrug therapy (CDEPT) and in combination with prodrug CB1954 or PR-104 showed significant antitumor efficacy.^14,15^ The promoter-5′ UTR sequences and *nmeNTR* coding sequence were cloned into pGG2121, as described previously and confirmed by PCR screening and Sanger sequencing. The resulting series of pGG2121-P*romoter*-*nmeNTR* plasmids were transformed into *E. coli* S17-1 and then conjugated into *C. sporogenes*-NT. After confirmation of *Clostridium* transconjugants by Sanger sequencing, 7 h subcultures were harvested and the menadione nitroreductase assay (Figure 5a) was performed. As shown in Figure 5b, the menadione nitroreductase and glucuronidase activities showed a good linear correlation among these eight promoters-5′ UTR in *C. sporogenes*-NT, with a coefficient of determination *R*^2^ of 0.9372. We anticipate this cancer delivery vehicle *C. sporogenes*-NT with the highest *nmeNTR* gene expression to increase the CDEPT antitumor activity, compared to previously published results.^15^ As a consideration, compared to the GusA assay, menadione nitroreductase assay is more expensive in materials and more demanding for the use of suitable equipment. It implies that conducting GusA is more convenient as a lab-based reporter system for expression validation in *Clostridium* spp.

### Alternative Start Codons Further Tune Gene Expression between Clostridia and *E. coli*

An ideal promoter for *Clostridium* metabolic engineering would have two critical characteristics: low expression in the *E. coli* cloning strain and constitutively high expression in clostridia.^70^ In our promoter-5′ UTR library, the range of GusA expression in *E. coli* was considerably less than in *Clostridium* species, exhibiting only a 3-fold change between the weakest (P*syn*_fU) and strongest (miniP*4*_tU) promoters-5′ UTR (Figure S2). In addition, all of the promoter-5′ UTR sequences in our study resulted in a high gene expression level in *E. coli*. In the context of gene products that are toxic to *E. coli*, this may prevent cloning of the gene of interest. To further tune the gene expression between clostridia and *E. coli*, we set out to investigate the effect of the start codon sequence on heterologous gene expression in *E. coli*, *C. sporogenes*, and *C. butyricum*. Previously, non-AUG start codons are annotated as initiation codons in 69 bacterial genomes including GUG, UUG, CUG, AUC, AUU, and AUA, from which translation initiated at 0.1–100% of AUG in *E. coli*.^32^ Inspired by this finding, we changed the AUG start codon of *gusA* gene with six alternative start codons (GUG, UUG, CUG, AUC, AUU, and AUA) and used the promoter-5′ UTR sequences of native P*fdx*_fU and the P*fdx*_tU to drive the expression, obtaining a series of vectors pGG2121-P*fdx*_fU-non-AUG*gusA* and pGG2121-P*fdx*_tU-non-AUG*gusA*.

**Figure 6:**
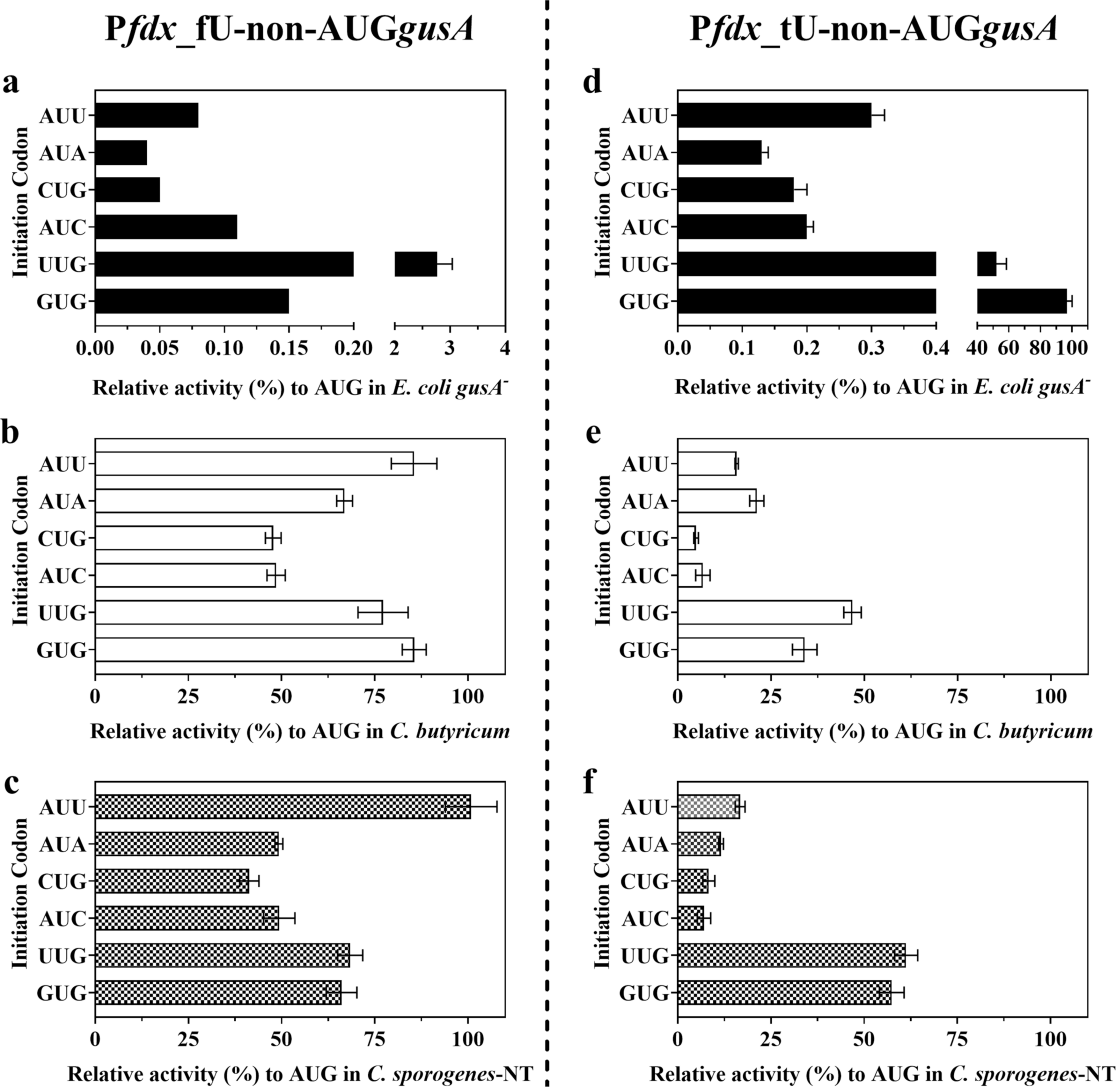
Alternative start codons further tune the GusA expression between *E. coli* and clostridia. Expression was driven by the native P*fdx*_fU and compared to the GusA specific activity of AUG start codon, the relative activity of GusA from non-AUG start codons in (a) *E. coligusA*^–^, (b) *C. butyricum*, and (c) *C. sporogenes*-NT. Expression was driven by the promoter-5′ UTR P*fdx*_tU and compared to the GusA specific activity of AUG start codon, the relative activity of GusA from non-AUG start codons in (d) *E. coligusA*^–^, (e) *C. butyricum*, and (f) *C. sporogenes*-NT. Data represent the mean ± s.d. of three biological replicates.

Driven by the native P*fdx*_fU, GusA expression initiated from all six alternative start codons showed only a 0.04–3% activity of AUG in *E. coli* (Figure 6a), while in *C. butyricum* and *C. sporogenes*, GusA expression showed 45–85% and 40–100% activities of AUG, respectively (Figure 6b,c). These results demonstrate that alternative start codons affect gene expression, but that the effect is radically different in *E. coli* and *Clostridium* spp. Similarly, driven by the P*fdx*_tU and initiated from the four alternative start codons (AUU, AUA, CUG, and AUC), GusA expression showed only a 0.1–0.3% activity of AUG in *E. coli* (Figure 6d), while a 5–20% activity of AUG was seen in *C. butyricum* (Figure 6e) and a 7–17% activity of AUG was seen in *C. sporogenes* (Figure 6f). These results also suggest that different 5′ UTRs (tU and fU) can affect translation initiation. In addition, driven by the P*fdx*_tU, GusA expression initiated from GUG and UUG showed 96 and 50% activities of AUG in *E. coli* (Figure 6d), which is significantly different from their relative activity driven by the native P*fdx*_fU in *E. coli* (Figure 6a). We reasoned that GUG or UUG start codon might tend to form a translation initiation signal with the Shine–Dalgarno sequence of tU,^71^ and between the Shine–Dalgarno sequence and the start codon, the spacer of fU (TGTGTTACAT) could create out-of-frame canonical start codons.^72^ The regulation of translation initiation from the 5′ UTR is complicated, and it is likely to vary between species. However, the aforementioned results indicate that translation initiation from these alternative start codons (AUU, AUA, CUG, and AUC)—at least in the two contexts that were tested—is more efficient in clostridia than in *E. coli*. In bacteria, translation initiation requires initiation factors (IF1, IF2, and IF3) and the initiator tRNA (fMet-tRNA^fMet^), in which IF3 increases the accuracy of initiator tRNA selection, monitoring codon–anticodon interactions.^73,74^ We reasoned that compared to *E. coli*, the initiation factors in clostridia might be less stringent in selecting the initiator tRNA. Another explanation is that the initiator tRNA may be more abundant in clostridia than in *E. coli*. Therefore, we encourage reconsidering gene annotations in *Clostridium* genomes and further exploration of translation initiation in clostridia. Overall, combined with our promoter-5′ UTR library before, the library of alternative start codons could further tune the gene expression between clostridia and *E. coli*.

### Tune the Expression of *sacB* Gene from *B. subtilis* as a Negative Marker in *C. butyricum*

**Figure 7:**
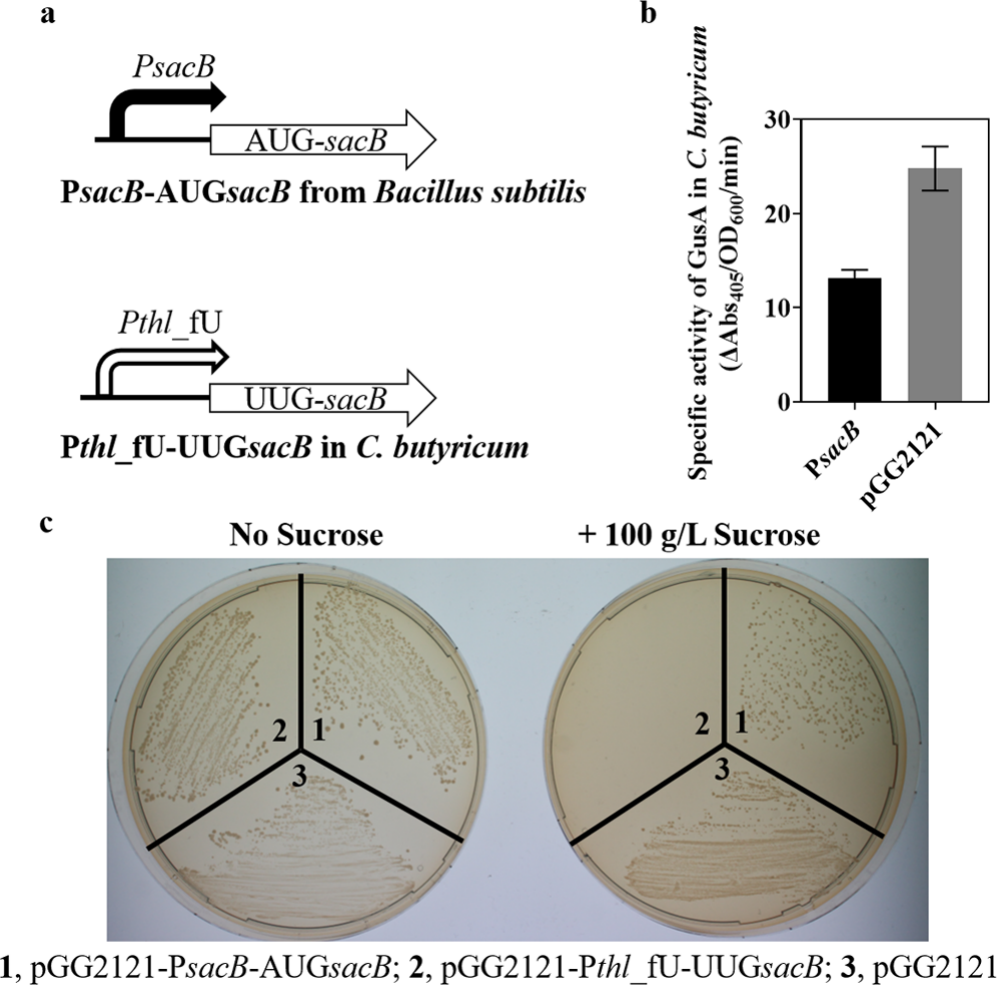
*sacB* gene from *B. subtilis* as a negative marker in *C. butyricum*. (a) The schema of *sacB* expression cassettes. (b) Strength of promoter P*sacB* in *C. butyricum* by glucuronidase assay, vector pGG2121 as a control. (c) Testing of sucrose sensitivity in *C. butyricum* containing vectors pGG2121-P*sacB*-AUG*sacB*, pGG2121-P*thl*_fU-UUG*sacB*, and pGG2121. Data represent the mean ± s.d. of three biological replicates.

To demonstrate the value of our libraries of promoters-5′ UTR and alternative start codons, we set out to tune the expression of the *sacB* gene for expression in *C. butyricum*. The *sacB* gene originates from *B. subtilis* and encodes the secreted levansucrase that converts sucrose into levans, which have toxic effects on bacterial cells.^75,76^ The *sacB* gene has been used as a negative selection marker to screen for double-crossover events in *E. coli* since the early 1990s and in numerous Gram-negative bacteria subsequently.^77^ Although certain species, such as *Corynebacterium glutamicum* and species of genus *Mycobacterium*, show sucrose sensitivity under *sacB* expression,^78^ to date there is no report of using *sacB* as a negative selection marker in clostridia. Thus, *sacB* with its native promoter P*sacB* (Figure 7a) was amplified from *B. subtilis* strain 168 and cloned into pGG2121 by Golden Gate assembly, obtaining vector pGG2121-P*sacB*-AUG*sacB*. As shown in Figure 7c, *C. butyricum* containing vector pGG2121-P*sacB*-AUG*sacB* was not sensitive to 100 g/L of sucrose. The native P*sacB* promoter was cloned into our GusA reporter system to determine its function in *C. butyricum*. Very low GusA activity was observed in *C. butyricum* (Figure 7b), suggesting that the native *B. subtilis* expression cassette (P*sacB*-AUG*sacB*) would not be functional in *C. butyricum*. Thus, we selected the P*thl*_fU, shown to have a medium–high strength in our promoter-5′ UTR library of *C. butyricum* (Figure 4c), to express the *sacB* gene. Golden Gate assembly with the P*thl*_fU and *sacB* fragments yielded very few colonies on cloning plates. In addition, the clones that did grow contained SNPs or an insertion sequence (Table S4). This cloning difficulty is similar to previous reports in *ilvB* (encoding acetohydroxyacid synthase) clones of *E. coli*.^79^ The P*thl*_fU can express a significant GusA level in *E. coli* (Figure S2), and cloning the native *sacB* expression cassette can result in fragility of the *E. coli* envelopes, as previously demonstrated.^80^ Thus, we reasoned that the high level of *sacB* expression by the P*thl*_fU increased the toxic effect in *E. coli* and resulted in cloning difficulties of the expression cassette P*thl*_fU-AUG*sacB*. To selectively downregulate *sacB* expression in *E. coli*, we used the UUG start codon for translation initiation, previously shown to reduce GusA expression to a 3% activity of AUG in *E. coli*, driven by the native P*fdx*_fU (Figure 6a). This change enabled us to assemble and clone the *sacB* expression vector without issues. *C. butyricum* containing vector pGG2121-P*thl*_fU-UUG*sacB* showed significant sensitivity to sucrose, in line with previous reports in other bacteria (Figure 7c). This result marks the creation of a novel negative selection marker for use in *Clostridium* species and demonstrates the benefit of rationally designed heterologous gene expression.

In conclusion, in this study we first report the construction of a modular promoter-5′ UTR library in different *Clostridium* spp. and validated the library using two enzymatic assays. Reporter expression from native and rationally designed synthetic promoters was evaluated with two native 5′ UTRs and exhibited a wide range of strengths in two species, *C. sporogenes*-NT and *C. butyricum*. Second, a library of alternative start codons enabled species-specific control of gene expression in *E. coli* and clostridia. Third, the results of the two libraries were used to create a novel negative selection marker in *C. butyricum*. Our results underline the crucial importance and possibilities of fine-tuning native promoters, the UTRs as well as the start codons, to achieve a broad dynamic range of gene expression for *Clostridium*-mediated applications.

## Materials and Methods

### Bacterial Strains and Growth Conditions

Details of the used strain are all listed in Table 1. Three *E. coli* strains (10-β, *gusA*^-^, and S17-1) in this study were used for the cloning, GusA assay, and conjugation, respectively. In addition, *E. coli* strains containing plasmids (Table S1) were grown at 37 °C in LB broth supplemented with 12.5 μg/mL of chloramphenicol or on LB agar plates supplemented with 25 μg/mL of chloramphenicol. No-toxic strain of *C. sporogenes* NCIMB 10696 was created previously by deleting the putative Streptolysin S (SLS) operon.^12^*C. butyricum*-type strain DSM 10702 was purchased from DSMZ-German Collection. These *Clostridium* strains in this study were grown in peptone yeast thioglycolate media^12^ with the addition of 10 g/L of d-glucose (PYTG), supplemented with d-cycloserine (250 μg/mL) and thiamphenicol (15 μg/mL) when necessary. Then, the culture was incubated at 37 °C in an anaerobic cabinet (MG1000 Mark II, Don Whitley, U.K.; 80% N_2_, 10% CO_2_, 10% H_2_).

**Table 1:** Strains and Plasmids Used in This Work

strains	description	sources
*E. coli* 10-β	high-efficiency strain ideal for cloning	C3019, NEB
*E. coligusA*^–^	*E. coli* JW1609 with *gusA* knockout mutant	OEC4987-200827007, Horizon Discovery Ltd.,^50^
*E. coli* S17-1	conjugative donor strain	ATCC 47055
*C. sporogenes*-NT	no-toxic strain of *Clostridium sporogenes* NCIMB 10696 by deleting the putative Streptolysin S (SLS) operon	(12)
*C. butyricum*	type strain *Clostridium butyricum* DSM 10702	DSMZ-German Collection

plasmids	description	sources
pMTL82151	*E. coli*–*Clostridium* shuttle vector, Cm^r^, Gram^–^ replicon ColE1	University of Nottingham^2^
pMTL82121	*E. coli*–*Clostridium* shuttle vector, Cm^r^, Gram^–^ replicon p15a	University of Nottingham^2^
pGG2151	golden gate assembling vector based on pMTL82151	this work
pGG2121	golden gate assembling vector based on pMTL82121	this work
pMiniT 2.0	an *E. coli* plasmid cloning vector in PCR Cloning Kit, Amp^r^	E1202, NEB
pMiniT 2.0-*gusA*	*gusA* fragment with golden gate *Bsa*I sites in pMiniT 2.0	this work
pRPF185	*E. coli*–*C. difficile* shuttle vector containing a codon-optimized gene *gusA* for *C. difficile*	106367, Addgene,^46^
pGG2121-*gusA*	pGG2121 ligating only with reporter GusA	this work
pGG2121-P*romoter*-*gusA*	pGG2121 ligating with promoters and reporter GusA	this work
pGG2121-P*romoter*-*nmeNTR*	pGG2121 ligating with promoters and NmeNTR	this work
pGG2121-P*fdx*_fU-non-AUG*gusA*	pGG2121 ligating with native P*fdx*_fU and *gusA* gene with alternative start codons	this work
pGG2121-P*fdx*_tU-non-AUG*gusA*	pGG2121 ligating with promoter-5′ UTR P*fdx*_tU and *gusA* with alternative start codons	this work
pGG2121-P*sacB*-AUG*sacB*	pGG2121 ligating with promoter P*sacB* and *sacB* gene from *B. subtilis* strain 168	this work
pGG2121-P*thl*_fU-UUG*sacB*	pGG2121 ligating with promoter-5′ UTR P*thl*_fU and *sacB* gene with the UUG start codon	this work

### Plasmid Construction and Transformation

Details of the used plasmids are all listed in Table 1, and primers are all listed in the Supporting information (Table S1). All of the plasmids constructed in this study were confirmed by PCR screening of M13-F/R and Sanger sequencing. The shuttle vectors pMTL82151 and pMTL82121 were obtained from Prof. Minton (SBRC, University of Nottingham). The vector pMTL82151/pMTL82121 was digested by *Mre*I/*Nhe*I to remove the terminator CD0164 and multiple cloning site (MCS), and the ∼4 kb fragment was purified as the backbone. The fragment of terminator *tyrS* was amplified by primers with Golden Gate *Bsm*BI sites. In addition, new MCS was amplified by primers with Golden Gate *Bsa*I sites, universal primer pairs M13-F/R sequences, and Golden Gate *Bsm*BI sites. According to the Golden Gate assembly protocol (*Bsm*BI-v2) [E1602, New England Biolabs (NEB)], these three fragments were ligated and transformed into *E. coli* 10-β, obtaining the Golden Gate assembly plasmid pGG2121. Two primers with Golden Gate *Bsa*I sites were designed to amplify *gusA* gene using pRPF185 as a template. To enable the rapid and precise cloning of the GusA reporter system, the ORF of *gusA* gene was blunt-cloned into vector pMiniT 2.0 by PCR Cloning Kit (E1202, NEB). The resulting plasmid pMiniT 2.0-*gusA* (Figure 1) was sequence-confirmed and served as a template for further molecular construction. The fragments of promoters and 5′ UTRs were amplified by primers with Golden Gate *Bsa*I sites, and then according to the Golden Gate assembly Protocol (*Bsa*I-HFv2) (E1601, NEB), *gusA* fragment in pMiniT 2.0-*gusA* and promoters were ligated into plasmid pGG2121. Similarly, *nmeNTR* fragment^14^ and *sacB* fragment were amplified and ligated with selected promoters into plasmid pGG2121 by Golden Gate assembly. Plasmids were transformed by heat shock into *E. coli* strains and transferred to *Clostridium* strains by conjugation, as described previously.^2^

### Glucuronidase Reporter Assay

The glucuronidase (GusA) activity in *E. coligusA*^*–*^ and *Clostridium* spp. was evaluated as described by Paweł and John.^28^ The overnight culture of strains containing GusA expression plasmids was inoculated into a fresh PYTG media (1:100). As OD_600_ of the cultures grow to about 1.0, samples of 1.5 mL were harvested by centrifugation and the pellets were frozen at −80 °C. Similarly, the pellet testing was administrated as described and the specific activity of GusA was calculated in the unit of ΔAbs_405_/OD_600_/min by dividing the absorbance at 405 nm by sample OD_600_ nm and incubation time.

### Menadione Nitroreductase Assay

According to Knox et al.,^81^ the nitroreductase (NTR) activity in *C. sporogenes* was determined using menadione (M9429, Sigma-Aldrich) as an enzymatic substrate and bovine cytochrome *c* (C3131, Sigma-Aldrich) as an electron acceptor and also a colorimetric chemical. Briefly, the overnight culture of strains containing NmeNTR expression plasmids was inoculated into a fresh PYTG media (1:100). After 7 h, samples of 2 mL were harvested by centrifugation and the pellets were frozen at −80 °C. As for stock solutions, 1 mM of menadione was made up in dimethyl sulfoxide (DMSO) and stored at room temperature. The other solutions of 10 mM of NADH (N4505, Sigma-Aldrich) and 700 μM of bovine cytochrome *c* were dissolved in 10 mM of tris–HCl buffer, pH 7.5 and stored at −20 °C. Before testing, the pellets were lysed and the soluble proteins inside were extracted in 300 μL of BugBuster Master Mix/protease solution [Dissolving one of cOmplete, Mini, EDTA-free Protease Inhibitor Tablets (11836170001, ROCHE) in 2 mL of BugBuster Master Mix (71456-3, Millipore) and then diluting 1:25], which then were diluted in 10 mM of tris–HCl buffer. Moreover, using a flat-bottom 96-well plate, 150 μL/wells of reaction master mix (200 μL of 1 mM menadione, 2 mL of 10 mM NADH, 2 mL of 700 μM bovine cytochrome *c*, and 10.8 mL of 10 mM tris–HCl buffer, pH 7.5) was prepared and preheated at 37 °C for about 10 min in a multimode microplate reader (iD3, SpectraMax). Then, 10 μL of diluted lysates with 40 μL of 10 mM tris–HCl buffer was added into wells at 37 °C. During the incubation, the increase of absorbance at 550 nm for 1 min was recorded and the rate divided by the volume (in mL) of lysate used was 14.79 (the extinction coefficient of cytochrome *c* in cm^–1^ μM^–1^), obtaining the menadione nitroreductase activity expressed in units per mL (U/mL). Finally, the menadione nitroreductase activity tested above was normalized to units per g (U/mg) by dividing the total protein concentration (mg/mL) inside the used lysate, which was determined as the BCA Protein Assay Kit (23225, Thermo Scientific) described.

## Supporting Information Available

Summary of promoter-5′ UTR library in *C. sporogenes*-NT and *E. coli*; list of primer sequences; list of promoter-5′ UTR sequences; list of aligned promoter sequences; and sequences of *sacB* expression cassettes (PDF)

## Notes

P.L. reports, within the submitted work, presenter fee and/or reimbursement of travel costs/consultancy fee and/or in kind main power contribution from Benelux Health Ventures, Merck, BMS, Convert Pharmaceuticals, and LivingMed Biotech. P.L. has minority shares in the company LivingMed Biotech and Convert Pharmaceutical. A.M.K. is an employee of Exomnis Biotech. J.T. has minority shares in LivingMed Biotech.

## Abbreviations

ABE: acetone–butanol–ethanol
CDEPT: clostridia-directed enzyme prodrug therapy
PCEs: prodrug converting enzymes
5′ UTR: 5′ untranslated region
GFP: green fluorescent protein
ORF: open reading frame
SNPs: single nucleotide polymorphisms
OD: optical density
PCR: polymerase chain reaction
tU: 5′ untranslated region of P*thl*
fU: 5′ untranslated region of P*fdx*
SLS: Streptolysin S
PYTG: peptone yeast thioglycolate media with the addition of 10 g/L of d-glucose
BCA: bicinchoninic acid
