# Supplementary material for: Heterologous Gene Regulation in Clostridia: Rationally Designed Gene Regulation for Industrial and Medical Applications
Source: ACS Synth Biol. 2022 Oct 20;11(11):3817–28. doi: 10.1021/acssynbio.2c00401 (PMC9680021; doi:10.1021/acssynbio.2c00401)
Supplement: Supplementary file 1 — sb2c00401_si_001.pdf [file sb2c00401_si_001.pdf]

Supporting Information

Heterologous Gene Regulation in Clostridia: Rationally Designed Gene Regulation for Industrial and Medical Applications

Yanchao Zhang[1*], Tom S. Bailey[1], Aleksandra M. Kubiak[1, 2], Philippe Lambin[1], and Jan Theys[1]

[1] The M-Lab, Department of Precision Medicine, GROW - School of Oncology and Reproduction, Maastricht University, 6229 ER Maastricht, the Netherlands

[2] Exomnis Biotech BV, Oxfordlaan 55, 6229 EV Maastricht, the Netherlands

Corresponding Author
Yanchao Zhang, Email: yanchao.zhang@maastrichtuniversity.nl

Figure S1. Summary of promoter-5' UTR library in *C. sporogenes*-NT.

Figure S2. Summary of promoter-5' UTR library in *E. coli gusA*$^-$.

Table S1. Primers used in the study.

Table S2. Collection of promoters-5' UTR sequences in the study.

Table S3. Sequences of aligned promoter in PePPER and Weblogo.

Table S4. Sequences of *sacB* expression cassettes.

Figure S1.

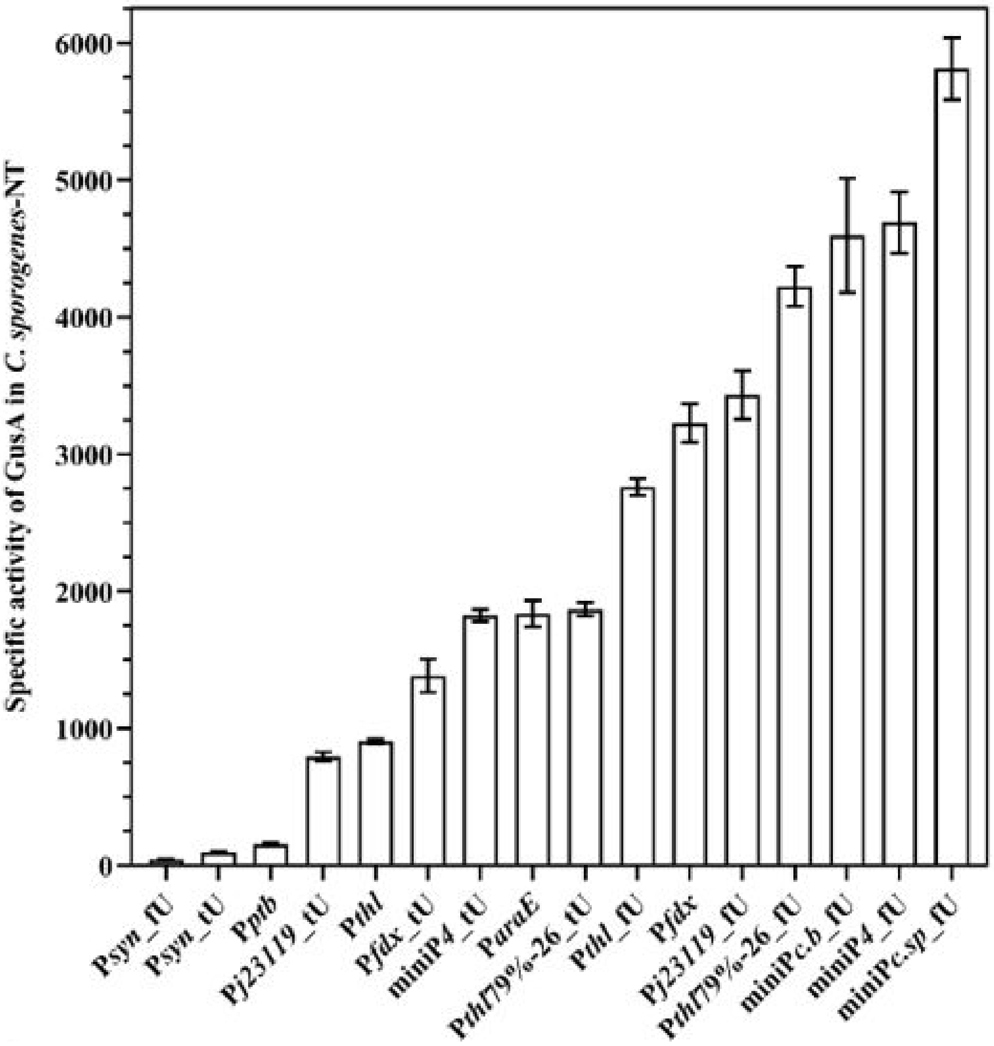

Figure S1. Summary of promoter-5' UTR library in *C. sporogenes*-NT. Data represent the mean ± s.d. of three biological replicates.

Figure S2.

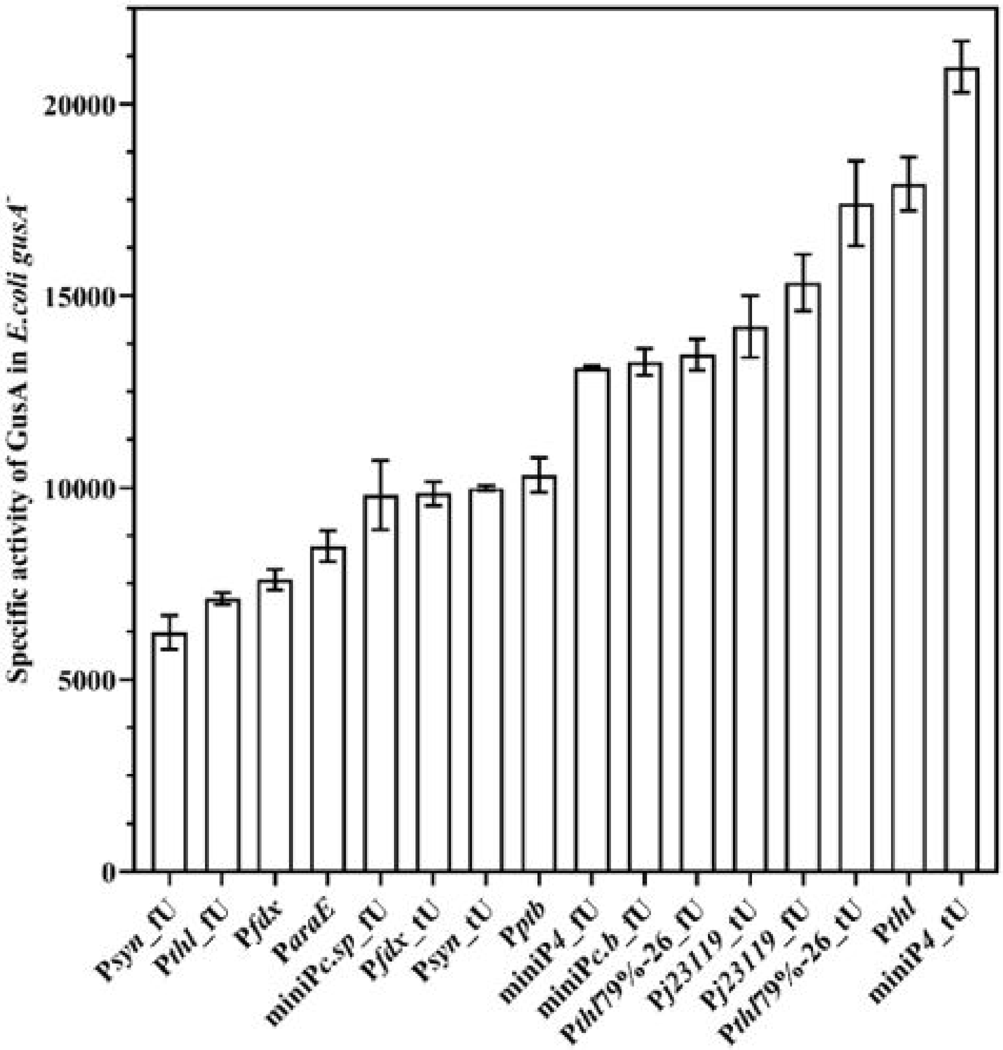

Figure S2. Summary of promoter-5' UTR library in *E. coli gusA*⁻. Data represent the mean ± s.d. of three biological replicates.

Table S1. Primers used in the study. Restriction sites are underlined.

Primers	5' - 3' Sequences	Description
BsmBI-MreI-F	TATACGTCTCACCGGCGTAACAGATGAGGGC	Amplification of fragment with terminator *tyrS* from pMTL82121
BsmBI-TtyrS-R	TATACGTCTCAACACGAAGGGACGATTGATTGATTATCCTGCAGGGGGCCC	
BsmBI-MCS-F	TATACGTCTCTGTGTAAACGAAGGGGCGTTTTTATTTCAGGAAACAGCTATGACCGCGGCCGCTGTATCCATGAGACC	Amplification of new MCS fragment with *Bsa*I sites and M13F/R sequences from pMTL82121
BsmBI-MCS-R	TATACGTCTCGCTAGTGTAAAACGACGGCCAGTGCCAAGCTTGCATGTCTGGAGACCTCG	
M13-F	GTAAAACGACGGCCAGTG	Screening primer for pCLOS_GG2121
M13-R	CAGGAAACAGCTATGACCG	
BsaI-gusA-F	TATAGGTCTCAATGTTACGTCCTGTAGAAACCCC	Amplification of *gusA* fragment from pRPF185
BsaI-gusA-R	TATAGGTCTCAGTCTTCATTGTTTGCCTCCCTGC	
BsaI-Pthl-F	TATAGGTCTCGTCCATTTTTAACAAAATATATTGATAAAAATAATAATAGTGG	Amplification of P*thl* from *C. acetobutylicum* ATCC 824 to ligate reporter GusA
BsaI-Pthl_gusA-R	TATAGGTCTCGACATTCTAACTAACCTCCTAAATTTTGATAC	
BsaI-Pptb-F	TATAGGTCTCGTCCAATAAGTCAGCAGAAAGTATAATGAG	Amplification of P*ptb* from *C. acetobutylicum* ATCC 824 to ligate reporter GusA
BsaI-Pptb_gusA-R	TATAGGTCTCGACATTGGTCGTACACTCCCTTTTAC	
BsaI-ParaE-F	TATAGGTCTCGTCCATTTATATTTAGTCCCTTGCCTTGC	Amplification of P*araE* from *C. acetobutylicum* ATCC 824 to ligate reporter GusA
BsaI-ParaE_gusA-R	TATAGGTCTCGACATGAAAACTCCTCCTTAAGATTTATATATG	
BsaI-Pfdx-F	TATAGGTCTCGTCCATATAAAAATTACTTTAAAAATTAATAAAAACATGGT	Amplification of P*fdx* from *C. sporogenes* NCIMB 10696 to ligate reporter GusA
BsaI-Pfdx_gusA-R	TATAGGTCTCGACATATGTAACACACCTCCTTAAAAAT	
BsaI-Pthl_cut-R	TATAGGTCTCCTACGTTAATTATACCCACTATTATTATTTTTATCAATATATTTTGTTAAAAATGGACGAGACC	Amplification with primer BsaI-Pthl-F for P*thl* without 5'UTR
BsaI-Pthl_UTR-F	TATAGGTCTCCGTTGTTAGAGAAAACGTATAAATTAGG	Amplification with primer BsaI-Pthl_gusA-R for 5'UTR of P*thl* to ligate reporter GusA
BsaI-Pfdx_cut-R	TATAGGTCTCGCAACATTTATATTTTACCATGTTTTTATTAATTTTTAAAGTAATTTTTATATGGACGAGACC	Amplification with primer BsaI-Pfdx-F for P*fdx* without 5'UTR
BsaI-Pfdx_UTR-F	TATAGGTCTCGCGTATAAAGTTGTGTAATTTTTAAGGAGGTGTGTTACATATGTCGAGACC	Amplification with primer BsaI-Pfdx_gusA-R for 5'UTR of P*fdx* to ligate reporter GusA
BsaI-Pj23119_tU_cut-F	TATAGGTCTCGTCCATTGACAGCTAGCTCAGTCCTAGGTATAATGTTGGGAGACCTATA	Primes annealing for P*j23119*_tU without 5'UTR of P*thl*
BsaI-Pj23119_tU_cut-R	TATAGGTCTCCCAACATTATACCTAGGACTGAGCTAGCTGTCAATGGACGAGACCTATA	
BsaI-Psyn_tU_cut-F	TATAGGTCTCGTCCATTGACATGGGCTCACGAGAGCCTCTACTATAATATTGTTGGGAGACCTATA	Primes annealing for P*syn*_tU without 5'UTR of P*thl*
BsaI-Psyn_tU_cut-R	TATAGGTCTCCCAACAATATTATAGTAGAGGCTCTCGTGAGCCCATGTCAATGGACGAGACCTATA	
BsaI-Pthl7926_tU_cut-F	TATAGGTCTCGTCCACTTTTAACAAAATATATTGATAAAAATTATAATAGTGTGTATAATTAAGTTGGGAGACCTATA	Primes annealing for P*thl*79%-26_tU without 5'UTR of P*thl*

BsaI-Pthl7926_tU_cut-R	TATAGGTCTCCCAACTTAATTATACACACTATTATAATTTTTATCAATATATTTTGTTAAAAGTGGACGAGACCTATA	
BsaI-miniP4_ tU_cut-F<br>BsaI-miniP4_ tU_cut-R	TATAGGTCTCGTCCATTGACAAATTTATTTTTTAAAGTTAAAATTAAGTTGGGAGACCTATA<br>TATAGGTCTCCCAACTTAATTTTAACTTTAAAAAATAAATTTGTCAATGGACGAGACCTATA	Primes annealing for miniP*4*_tU without 5'UTR of P*thl*
BsaI-Pj23119_fU_cut-F<br>BsaI-Pj23119_fU_cut-R	GGTCTCGTCCATTGACAGCTAGCTCAGTCCTAGGTATAATATAAATCGTAGGAGACC<br>GGTCTCCTACGATTTATATTATACCTAGGACTGAGCTAGCTGTCAATGGACGAGACC	Primes annealing for P*j23119*_fU without 5'UTR of P*fdx*
BsaI-Psyn_fU_cut-F<br>BsaI-Psyn_fU_cut-R	GGTCTCGTCCATTGACATGGGCTCACGAGAGCCTCTACTATATAAATCGTAGGAGACC<br>GGTCTCCTACGATTTATATAGTAGAGGCTCTCGTGAGCCCATGTCAATGGACGAGACC	Primes annealing for P*syn*_fU without 5'UTR of P*fdx*
BsaI-Pthl7926_fU_cut-F<br>BsaI-Pthl7926_fU_cut-R	GGTCTCGTCCACTTTTAACAAAATATATTGATAAAAATTATAATAGTGTGTATAATATAAATCGTAGGAGACC<br>GGTCTCCTACGATTTATATTATACACACTATTATAATTTTTATCAATATATTTTGTTAAAAGTGGACGAGACC	Primes annealing for P*thl*79%-26_fU without 5'UTR of P*fdx*
BsaI-miniP4_ fU_cut-F<br>BsaI-miniP4_ fU_cut-R	GGTCTCGTCCATTGACAAATTTATTTTTTAAAGTTAAAATATAAATCGTAGGAGACC<br>GGTCTCCTACGATTTATATTTTAACTTTAAAAAATAAATTTGTCAATGGACGAGACC	Primes annealing for miniP*4*_fU without 5'UTR of P*fdx*
BsaI-miniPc.sp_fU_cut-F<br>BsaI-miniPc.sp_fU_cut-R	GGTCTCGTCCATTGACAAAAAAAATTAAATATTATATAATATAAATCGTAGGAGACC<br>GGTCTCCTACGATTTATATTATATAATATTTAATTTTTTTTGTCAATGGACGAGACC	Primes annealing for miniP*c.sp*_fU without 5'UTR of P*fdx*
BsaI-miniPc.b_fU_cut-F<br>BsaI-miniPc.b_fU_cut-R	GGTCTCGTCCATTGACTTAATTTAAATTTAATTATATAATATAAATCGTAGGAGACC<br>GGTCTCCTACGATTTATATTATATAATTAAATTTAAATTAAGTCAATGGACGAGACC	Primes annealing for miniP*c.b*_fU without 5'UTR of P*fdx*
BsaI-nmeNTR-F<br>BsaI-nmeNTR-R	GGTCTCGATGACAGTATTATCTAAAGAACAAGTATTATC<br>GGTCTCAGTCTTTAAGCCCATATTACAGTCTCTTC	Amplification of *nmeNTR* fragment
BsaI-Pptb_NTR-R	GGTCTCGTCATTGGTCGTACACTCCCTTTTAC	Amplification of P*ptb* with primer BsaI-Pptb-F to ligate NmeNTR
BsaI-Pfdx_NTR-R	GGTCTCGTCATATGTAACACACCTCCTTAAAAAT	Amplification of P*fdx* and P*thl*_fU with primer BsaI-Pfdx-F and BsaI-Pthl-F respectively to ligate NmeNTR
BsaI-Pthl_NTR-R	GGTCTCGTCATTCTAACTAACCTCCTAAATTTTGATAC	Amplification of P*thl* with primer BsaI-Pthl-F to ligate NmeNTR
BsaI-Pthl7926-F	GGTCTCGTCCACTTTTAACAAAATATATTGATAAAAATTATAATAGTGT	Amplification of P*thl*79%-26_tU and P*thl*79%-26_fU with primer BsaI-Pthl_NTR-R and BsaI-Pfdx_NTR-R respectively to ligate NmeNTR
BsaI-miniP4-F	GGTCTCGTCCATTGACAAATTTATTTTTTAAAGTTAAAATATAAATCGT	Amplification of miniP*4*_fU with primer BsaI-Pfdx_NTR-R to ligate NmeNTR

BsaI-miniPc.sp-F	GGTCTCGTCCATTGACAAAAAAAATTAAATATTATATAATATAAATCGT	Amplification of miniP*c.sp*_fU with primer BsaI-Pfdx_NTR-R to ligate NmeNTR
GusA_ATGcut-BsaI-F	GGTCTCATTACGTCCTGTAGAAACCC	With primer BsaI-gusA-R, amplification of *gusA* fragment without AUG start codon
Pfdx_fU-GTGgusA-BsaI-R<br>Pfdx_fU-TTGgusA-BsaI-R<br>Pfdx_fU-CTGgusA-BsaI-R<br>Pfdx_fU-ATCgusA-BsaI-R<br>Pfdx_fU-ATAgusA-BsaI-R<br>Pfdx_fU-ATTgusA-BsaI-R	GGTCTCGGTAACACATGTAACACACCTCCTTAAAAATTAC<br>GGTCTCGGTAACAAATGTAACACACCTCCTTAAAAATTAC<br>GGTCTCGGTAACAGATGTAACACACCTCCTTAAAAATTAC<br>GGTCTCGGTAAGATATGTAACACACCTCCTTAAAAATTAC<br>GGTCTCGGTAATATATGTAACACACCTCCTTAAAAATTAC<br>GGTCTCGGTAAAATATGTAACACACCTCCTTAAAAATTAC	With primer BsaI-Pfdx-F, amplification of fragments of P*fdx*_fU-non-AUG start codons to ligate *gusA* fragment without AUG start codon
Pfdx_tU-GTGgusA-BsaI-R<br>Pfdx_tU-TTGgusA-BsaI-R<br>Pfdx_tU-CTGgusA-BsaI-R<br>Pfdx_tU-ATCgusA-BsaI-R<br>Pfdx_tU-ATAgusA-BsaI-R<br>Pfdx_tU-ATTgusA-BsaI-R	GGTCTCGGTAACACTCTAACTAACCTCCTAAATTTTGATA<br>GGTCTCGGTAACAATCTAACTAACCTCCTAAATTTTGATA<br>GGTCTCGGTAACAGTCTAACTAACCTCCTAAATTTTGATA<br>GGTCTCGGTAAGATTCTAACTAACCTCCTAAATTTTGATA<br>GGTCTCGGTAATATTCTAACTAACCTCCTAAATTTTGATA<br>GGTCTCGGTAAAATTCTAACTAACCTCCTAAATTTTGATA	With primer BsaI-Pfdx-F, amplification of fragments of P*fdx*_tU-non-AUG start codons to ligate *gusA* fragment without AUG start codon
BsaI-PsacB-sacB-F<br>BsaI-SacB-R	GGTCTCATCCACACATATACCTGCCGTTCA<br>GGTCTCGGTCTTTATTTGTTAACTGTTAATTGTCCTTG	Amplification of *sacB* gene with P*sacB* from *Bacillus subtills* strain 168
BsaI-PsacB-gusA-R	GGTCTCGACATCGTTCATGTCTCCTTTTTTATG	Amplification of P*sacB* with BsaI-PsacB-sacB-F to ligate reporter GusA
BsaI-TTGsacB-F	GGTCTCTGAACATCAAAAAGTTTGCAAAAC	With primer BsaI-SacB-R, amplification of *sacB* with UUG start codon
Pthl_fU-TTGsacB-BsaI-R	GGTCTCGGTTCAAATGTAACACACCTCCTTAAAAATTAC	With primer BsaI-Pthl-F, amplification of P*thl*_fU to ligate *sacB* with UUG start codon

Table S2. Collection of promoters-5' UTR sequences in the study. -35 and -10 elements are highlighted in yellow; 5' UTRs are underlined; RBSs are highlighted in bold.

**Promoters**	**5' - 3' Sequences**
P*thl*	TTTTTAACAAAATATATTGATAAAAATAATAATAGTGGGTATAATTAAGTTGTTAGAGAAAACGTATAAATTAGGGATAAACTATGGAACTTATGAAATAGATTGAAATGGTTTATCTGTTACCCCGTATCAAAATTT**AGGAGG**TTAGTTAGA
P*fdx*	TATAAAAATTACTTTAAAAATTAATAAAAACATGGTAAAATATAAATCGTATAAAGTTGTGTAATTTTT**AAGGAGG**TGTGTTACAT
P*ptb*	GCGGCTGTGGATGGAGTTAAGTCAGCAGAAAGTATAATGAGAAAATATAAAATATAAATAATTTTCTAAAAAACTTAACTTCATGTGAAAAGTTTGTTAAAATATAAATGAGCACGTTAATCATTTAACATAGATAATTAAATAGT**AAAAGGGAG**TGTACGACCA
P*araE*	TTTATATTTAGTCCCTTGCCTTGCCTACAAGGGATTTCCTATTCCTTTCATTTACAATTCATACGTATAAAATCCAAATTTTTCTTGACATTTATACACATAAATATTATGATTTATATAGGTAATCGCTTTCATAAAATATATTACCCTTAGGAAATCAAATGATTATAAGTCATATATGAAAACGTTATATATAATTGATATGTTTACATTTGTAACTTAGATTTCTCTTTGATTTCCACATATATAAATCTT**AAGGAGGAG**TTTTC
P*thl*_fU	TTTTTAACAAAATATATTGATAAAAATAATAATAGTGGGTATAATTAACGTATAAAGTTGTGTAATTTTT**AAGGAGG**TGTGTTACAT
P*fdx*_tU	TATAAAAATTACTTTAAAAATTAATAAAAACATGGTAAAATATAAATGTTGTTAGAGAAAACGTATAAATTAGGGATAAACTATGGAACTTATGAAATAGATTGAAATGGTTTATCTGTTACCCCGTATCAAAATTT**AGGAGG**TTAGTTAGA
P*thl*79%_26_tU	CTTTTAACAAAATATATTGATAAAAATTATAATAGTGTGTATAATTAAGTTGTTAGAGAAAACGTATAAATTAGGGATAAACTATGGAACTTATGAAATAGATTGAAATGGTTTATCTGTTACCCCGTATCAAAATTT**AGGAGG**TTAGTTAGA
miniP*4*_tU	TTGACAAATTTATTTTTTAAAGTTAAAATTAAGTTGTTAGAGAAAACGTATAAATTAGGGATAAACTATGGAACTTATGAAATAGATTGAAATGGTTTATCTGTTACCCCGTATCAAAATTT**AGGAGG**TTAGTTAGA
P*j23119*_tU	TTGACAGCTAGCTCAGTCCTAGGTATAATGTTGTTAGAGAAAACGTATAAATTAGGGATAAACTATGGAACTTATGAAATAGATTGAAATGGTTTATCTGTTACCCCGTATCAAAATTT**AGGAGG**TTAGTTAGA
P*syn*_tU	TTGACATGGGCTCACGAGAGCCTCTACTATAATATTGTTGTTAGAGAAAACGTATAAATTAGGGATAAACTATGGAACTTATGAAATAGATTGAAATGGTTTATCTGTTACCCCGTATCAAAATTT**AGGAGG**TTAGTTAGA
P*thl*79%_26_fU	CTTTTAACAAAATATATTGATAAAAATTATAATAGTGTGTATAATATAAATCGTATAAAGTTGTGTAATTTTT**AAGGAGG**TGTGTTACAT
miniP*4*_fU	TTGACAAATTTATTTTTTAAAGTTAAAATATAAATCGTATAAAGTTGTGTAATTTTT**AAGGAGG**TGTGTTACAT
P*j23119*_fU	TTGACAGCTAGCTCAGTCCTAGGTATAATATAAATCGTATAAAGTTGTGTAATTTTT**AAGGAGG**TGTGTTACAT
P*syn*_fU	TTGACATGGGCTCACGAGAGCCTCTACTATATAAATCGTATAAAGTTGTGTAATTTTT**AAGGAGG**TGTGTTACAT
miniP*c.sp*_fU	TTGACAAAAAAAATTAAATATTATATAATATAAATCGTATAAAGTTGTGTAATTTTT**AAGGAGG**TGTGTTACAT
miniP*c.b*_fU	TTGACTTAATTTAAATTTAATTATATAATATAAATCGTATAAAGTTGTGTAATTTTT**AAGGAGG**TGTGTTACAT

Table S3. Sequences of aligned promoter in PePPER and Weblogo.

Genomes	5'-3' Promoter sequences
C. sporogenes NCIMB 10696	**363 promoters,**
(NZ_CP009225.1)	TATACTATAAAATCTCATTTAATTAAAAT
	TTGACAAAGAACAACAAATTTGATAAGAT
	TTGACAAATTGCAAAAAAGCAATTAAAAT
	TTGACATATAGCTATAGTATTTGTATAAT
	TTTAATTATAAACATAAATTTAATAAAAT
	TTGACAAAGTTTAATTATAATGCTATAAT
	TTGACATATATTCTTTTTATTGTTATAAT
	TTGAAATTATAGCTAAAATCAGTTATAAT
	TTGACAAAGAACAACAAATTTGTTAAGAT
	TTGACAAAGAACAACAAATTTGATAAGAT
	TTTAATATCTATTTATAATTATTTATAAT
	TTGACAAAGAACAACAAATTTGTTAAGAT
	TTGACAAAGAACAACAGATTTGATAAGAT
	TTGACAAGGAACAACAAATTTGTTAAGAT
	TTTACAGCGTATATAAATTAGTATATAAT
	TTTAATTTTAATATTGACAAGTTTAAAAT
	TTGACTTTTAATGTCATATTTGCTATTAT
	TTTACATTTAATATTTAATTTGTTAAAAT
	TTGACTAATTTAAAAATTTTAATTATAAT
	TTGACAGATTTAAATATACCGAATATAAT
	TTTAATAAATAAAGTTTATAAATTATAAT
	TTTACTAAATTTGCAATATTTGGTATAAT
	TTTACATAAACACTACCTTGTGCTACAAT
	TTGACAATAAAAAATTTGTATACTATAAT
	TTGAATATATAGATAACAGATGGTATAAT
	TTGACATTTTCTATATTTTAATGTATAAT
	TTGAAAATAATACAGAGTTTTTTTATAAT
	TAGACTATACGCTCTAACTTTTGTAAAAT
	TTGACATCATTGGAATTTTAATATATAAT
	TTGAATTAGGTATTATATTCTGTTATAAT
	TTTAATATTTTTTTGAAAAAATTTATAAT
	TTGAATGGAGTATAGTTAAATGATATAAT
	TTGAATTAAAATTATTAATAGTATATAAT
	TTGACAACTATATCTTAATATCGTAATAT
	TTGACAATATATATATCATAATATACAAT
	TTGAATTTATTTAGAATTTGAAATATAAT
	TAGACAGAGAACATGTTCAATGGTAAAAT
	TTGACATATGGCTTTGTATTATTTATAAT
	TTGACAACATAAATTTCCAAATATATAAT
	TTGACACAAATATTTATCTACTTTATAAT
	TTGACTTTAATCATAGTTAGAACTACAAT
	TTTACTCTAATAAATAACTTTACTATAAT
	TTGACAAATAAATATTTCTAGTGTAGAAT
	TTGAAATAAATAGATAATGTTGGTACAAT
	TTGACATAAAATGATTTTTTATATAATAT
	TTGAAATATAACATTTCTACGAATATAAT
	TTTAATAAATTCTTATTATATTATAAAAT
	TTTAATTTTTATTAAATTTACAATAAAAT
	TTTAATTTTAACATTTCAGATGTTAAAAT
	TTTAATTTTAACATCTGAAATGTTAAAAT
	TTTACTTTTATTTGAAGTTAAGGTAAAAT
	TATACTTTTTATATTGGTGGTGTTAAAAT
	TTGACTATATATGATATAAATACTATAAT
	TTTACATAATATATATAATTTTCTACAAT
	TTGAAATTAATTTTTAAAATGTATATAAT
	TTGAAATTAATACATATAATGTATACAAT
	TTTACAAATTCATTGCGCTAAAGTATAAT

	TTGAAAATTTTTTTTATTAATGATAAAAT
	TTGAAAATTAAACCTATTAAGTATAAAAT
	TTTACTCTACACTCTTATTTTTTTATAAT
	TTGACTTAAAATTTGGGCTGTAATATAAT
	TTTAATGTAAGTTACCATATATTTATAAT
	TTTAATATTATTTAAAAGAATACTAAAAT
	TTGAAATCTGGCAATTAATGATATAAAAT
	TTGACACAAAAATCCGTATAGTGTATAAT
	TTTAATTTATTATTAAACTTCTTTATAAT
	TATACTATAATAAAAAATTTTCATATAAT
	TTGACTTTTTTATATATTAATTTTAATAT
	TGGACTTAAATATAAAAAAGGGTTATAAT
	TTGACACTTATAATTATTAGTAATAAAAT
	TTGAAAAAAAGTTATTATTTATATACAAT
	TTGAAAAAAAGTTATTATTTATATACAAT
	TTTACTAATACTATAAAAGTAAATATAAT
	TTTACTTATAACAGTATTTAACCTATAAT
	TTGAATATATATTTTTATATGGATATAAT
	TTGACAATATACTTATACAATGATAATAT
	TTGACGGGTTTTCATTGTAAGTGTATAAT
	TTGACTTACCTATTATTAAATGTTATTAT
	TTGACTATTGAACAGATGTTCGATACAAT
	TTTACATAGGCTATAATATTTTTTATAAT
	TTGACTTTTATAATATAAATCTTTATAAT
	TTGACATATAATATTATAAATATTATAAT
	TTTACATGGCAAAACACAGTTAATATAAT
	TTGACATTGGAATTTTCCAGGTGTATTAT
	TTGACTTTTATTTTTTTATCTTTTATTAT
	TTGACAAAAATAGGCATCAATGATACAAT
	TTGACTTTCATCTTAAAATATGTTATACT
	TTTAATATTTAAAATACATACCATAAAAT
	TTGACTTTGGAATAAAAATTATATATAAT
	TTGACAAAATTATTCTAATACTCTATTAT
	TTTACTTTATGGTAACTTTATACTATAAT
	TTTACTTGGGAATTAATTCGTATTATAAT
	TTGACAAACAAAATAAATGAGCATATAAT
	TTGAAAGGGCCTAAAAAGAATGCTATAAT
	TAGACATTATATTTGTATTTCTGTATAAT
	TTTACATTAATATTTTTGTCATATATAAT
	TTTACAGGTCTTTTGAGTAAAGGTAAAAT
	TTTAATTATGTAAGTAAATTATATAAAAT
	TTTACAAGCTAAAATCACAAATTTAGAAT
	TTTAATATATGAATAATATTATCTATAAT
	TTGAAAAATAATTAAAATGAATGTATAAT
	TTTACAGATGTTACGGAGAACCGTACAAT
	TTGAATAAGAGGGTGCGAAAAAGTAAAAT
	TTTACATGCCACATAGACTTCTATACAAT
	TTGACTTATCTATTGGTAAATAATACTAT
	TTGAAAAATAAACCATACAAGTTTAAAAT
	TATACTTAGTAGTTTTTTTATTGTAAAAT
	TATACTTAGTAACCTATATCAATTATAAT
	TTTAATATAATTGATATAATGTTTAAAAT
	TTTAATTATATAAAGTATTTATTTATAAT
	TTGAAATTGATATTTATAATATGTACAAT
	TTGAAATTAAAGGTAATTGTGTATATAAT
	TTTACACAATCTCAATAATAAATTATAAT
	TTGAAAAATATGATAAAGTGGTTTATAAT
	TAGACACTTTCTTTTGTGTTTTATAAAAT
	TTGACATGGAAACAAAAATATATTAATAT
	TTGACAAAACAAATATTAAAGAGTAATAT

	TTTACAATAGGTTATAAATAAATTACAAT
	TTGAAATAATTCATCAATGGTGATATAAT
	TTGACAAATAATCTTTATGGACCTATAAT
	TAGACTATGATTTAGTATAAATTTATAAT
	TTGAAATTAATATTAGTAATGTATACAAT
	TTTACTATAACTGTTTTTTATAATAGAAT
	TTGACTTTCATTATTCTTGATGCTAAAAT
	TTGACATTAAATAGGCCTTGTTATAAAAT
	TTGAAAGTTTTGACAATAAATGATATAAT
	TTGACAAAATTATCTGAATATTGTATAAT
	TTGAAAATAATATTAATACATTATACAAT
	TTGACAGTTCATAAAATAAAATTTATAAT
	TTTACAATTTTAAGCCTGTATGCTATAAT
	TTTACGATATTTAATTTATATTTTAAAAT
	TTTAATACACCAATTTATTAATTTATAAT
	TTGACATAGAAATTTGCCATAATTATAAT
	TTGACTCAAAATAAATTTAACTTTAATAT
	TATACTAAATTAATTACACTTTCTATAAT
	TTGACATAAATTAATTAAAAGAATATAAT
	TTGACAATTATAACTAACAAAAGTAAAAT
	TTGACAGTAAATTTAAATCATGATATAAT
	TTTACTTAATATAATTTATCTGCTACAAT
	TTGAATTTTATGGTAATAAAATGTATAAT
	TTTACTATAATTCAATATTATTGTAAAAT
	TTTAATATATAAATAAATGTGAATAAAAT
	TTGACAATGGATGCTGTAGAAAGTAAAAT
	TTTACAACCTACCCTAATATTAATAAAAT
	TTTAATAAATTAAAATAATTATATATAAT
	TTGACAAGAAAACTTTAAAAGTATAATAT
	TTGAAAAAATATAAATGCAGTGTTATAAT
	TTTAATTAAAAATATAAAAATTTTAAAAT
	TTTACAAAAATTTATCATATTTTTAAAAT
	TATACTCATATTATACCTTTCAATAAAAT
	TTTAATATAAAGAACTATCCCTTTAAAAT
	TTTACAAAGTGTATTTTCTATATTAAAAT
	TTGACAAAATTTTCTTCATAAGTTAGAAT
	TTTAATTTTTATATTTGTGGTTTTAAAAT
	TTGACGGTAAATTATCGCAAATATATAAT
	TTTAATTTTTATATTTGCAGTGTTACAAT
	TTGACTTACAAACATGTGTTTGGTAAAAT
	TTTAATAGAATAAACAATAAGTGTATAAT
	TTGACATATGAAACTAAATGGTATAAAAT
	TTTACTTTTTTCTAATTTAAATATAAAAT
	TTGAATCTTTGAATATAATATTATATAAT
	TTGAATATTTTATCTTTTATTTATAAAAT
	TTTAATACAGTTATGTTATACTGTATAAT
	TTGAAATAAACTTTAGCCTTTAGTATAAT
	TTGAAAAACTAAATTAAATGTTGTATAAT
	TTGACTTAATGATAAAATTACGATAATAT
	TTGAATACTACATGGATAGAAAATATAAT
	TTTACATCTAACAACTTTTATTGTATAAT
	TTGAAATTCATAACTAATTATAATAAAAT
	TTGACAAGCCCTCTGGAAAGTTATATTAT
	TTGAATAGATTCCCATGATTCCATAAAAT
	TTTACTAAAAATATTTATCATAGTATAAT
	TTGACAGTAATAAAAAAAAATGGTAATAT
	TTTAATGCGTTTCTTATAAAAAATACAAT
	TTGAATTAATTATGCATTATATGTATAAT
	TTTACTTAAAGGGGTTTTTTATGTACAAT
	TTGAAAAATTTTATATTTAGTATTAAAAT

	TTGACTTTTGTTTATTGGAAGCATATACT
	TAGACTTTTAATAAATACTTTGGTAAAAT
	TTTACACACATTGTGCTTAATTTTATAAT
	TTGAAAAATGACTTAGAACAACTTATAAT
	TTTACAACTATAAATTTTTATTATATAAT
	TTGACAATGATATGCAAGTGTGGTAAGAT
	TTGACTTTATACAAAAGACAATATAATAT
	TTGACTGTATTATACTGAACTGATAAAAT
	TTGAAAAGGAAGTTTTATAGTTATATAAT
	TTGAAAGCGAATAATTATTCTTGTATAAT
	TAGACACATAAAAAAAGACATATTAAAAT
	TTGACTTTCTTTGACTTTTGTTTTATTAT
	TTGAAAAATGGAAACTATTTAAATACAAT
	TTGACAGGAATGGGGTGGAATTTTAAAAT
	TTGACTTAAATTTCATTAAGATTTATACT
	TTGAAAAAGTACTCTTAATAACATATAAT
	TTTAATTTTTATATTTAAAGTTCTAAAAT
	TTTAATATTTTTATTGTAACACTTATAAT
	TTGACACATAAAATATATTAAAATATAAT
	TTGACATATAAATTATATTAAAATATAAT
	TTTACAATTATAATTTTTTGGCATAAAAT
	TATACTATATAATTCATATGTTTTAAAAT
	TTGACACAAATTAACCAAGCATATACAAT
	TTGAAAAATGTATTATTATTATGTATAAT
	TTGACATGCATAAATATCCATTGTATAAT
	TTGACAGTATAATTAAAATAATTTACAAT
	TTGAAACAATCTCATCTTTAGTTTATAAT
	TTGAAAATGCCAAGTATATGTTATATAAT
	TTGAAAAATCCACTTGCATACTTTACAAT
	TTTACACAAAACACTTTTGGTATTATAAT
	TTTAATAATTAGTTGGGTAAGCGTAAAAT
	TTGACTTAGGATGTAAATCATGATATTAT
	TTGAATTTTATTTGTAATCTTTATATAAT
	TTTAATTTAATGAGTTAAAAATCTAAAAT
	TTGACGACGATTGGAAATTTTAATAAAAT
	TTTAATAAAACTACTCTGATAAATAAAAT
	TTTACAAATTTTACAAAAAGGATTAAAAT
	TATACTTACAGCATAATCTAATATATAAT
	TTTACGGCATTTTTATGTCGTAATAAAAT
	TTTAATTTACCTGGAAAAACTACTATAAT
	TTGACTTAAAAGGTTAATTCTGCTAATAT
	TTGAAAGCAATGAAAATGAACCATAAAAT
	TTTAATTTTTCCTTATAATAAATTATAAT
	TTTAATTGTTTTAATTATTAAATTATAAT
	TTGACGAAAATTCTAGAACTCTATATAAT
	TAGACTCTTTATTTTTATAATTTTATAAT
	TTGACAGATAAATACTTTATTGTTATAAT
	TTGACAAATTATAAAATACAGTATAATAT
	TTTAATTAAATAAATGAATTCCATAAAAT
	TTGACAACAATAAAAAAATAGGAGTAATAT
	TTGAAATTTATGTAAATATAATGTATAAT
	TTTAATTGCTGTTTCGAAATAAATATAAT
	TTGAAAAGTGCCCTTAAAGAAGGTAAAAT
	TTGACATGAAGATTGATATATATTACAAT
	TTGACTAATAAGACAAGGTTTGATAATAT
	TTGACAATTATTGTTAGTAAATGTATAAT
	TTTAATTTAATCAAAGCAACTTCTAAAAT
	TTGACAATACTTTTTTATATGATTATAAT
	TTTAATACTCTAATAGATCACATTATAAT
	TTTACTTATTGTCATATTAATTGTATAAT

	TTTAATACATACTTATAGTGCAATAAAAT
	TTTAATAAGGAATCCTTATTTATTACAAT
	TTGACATAATATTTTTCACCCTATATAAT
	TTGACAATTTTAATTAATAAGCCTATTAT
	TTGAATATTTGAACCATTAGCGGTATAAT
	TTGACTTCTTATACTACGTGTAGTATAAT
	TTTACTAATTATCGGTTTAACGATATAAT
	TTGAAATGTGTAGAAAAAAATGGTATAAT
	TTGACAATGGCAATTTTTTAATATAAAAT
	TTGACCCTAGTAAAAAAATAGTATATAAT
	TTTACAATTATATATTTATTTGTTATAAT
	TTGAAATTCATATTCTACTATGATAAAAT
	TTTAATTTAAAATAATTTGGAGGTATAAT
	TTGAATAAAAATTTTTACTATGGTAAAAT
	TTGACAGCTTTTTAATATTTTGATATTAT
	TTGACTTTTTAATATGCCCTTTATATAAT
	TTTAATATAAAATACAGATTTATTAAAAT
	TTTACTACTAAAAACTGCATATATAAAAT
	TTGAATAAATCCTTCATTTAATATAAAAT
	TTGACTAAATTACTATATTAATGTAATAT
	TTGACTTTTTTTGAGTATAATATTAAAAT
	TATACTTTTTAATAAATATGTCTTATAAT
	TTGACATAATGATAATTTTTATTTATAAT
	TTGACAGTTTTACTCAGGTTTGCTATTAT
	TATACTATTTTTAAAGCTATAAATAAAAT
	TAGACAAAAATATAAATAGTATGTACAAT
	TTGAAATATTAGTTGATTAAATTTAAAAT
	TTTAATATTTTCTTTTAATATGTTAAAAT
	TTGACAGTATTTATAGAATATATTAATAT
	TTGAATATTTATTATTTTATATGTATAAT
	TTGAAATTTAAAAGCTACGAATTTAAAAT
	TTGACAAAATATTGAATAAAAAATATAAT
	TTTACATATTCTTATAAATATTATATAAT
	TTGAAATTATCATAGTTACAATGTATAAT
	TTGAAAACGTAAACAAAAGATGGTAAAAT
	TTGAAAAAAATTACCAAAAGATGTATAAT
	TTGACATAGTTTTTGTAATATATTAAAAT
	TTGACAAAGAGAAAGGTGTATTATATTAT
	TTTAATGTTTTATAAAGAAAATTTAAAAT
	TTTAATATATTTTTAAAGCATAATAAAAT
	TTTACACAGAAAAGGGGGACTTTTAAAAT
	TTGACAAAATTCTATATAGAGAATAATAT
	TTGACTAATTCATGAATATAGGGTATTAT
	TTGACAAAAGTATGTAGTAACAATAAAAT
	TTTACAAAAAAAAAATTAAAGTATATAAT
	TTTACAGGAATTTAAATTATATCTAAAAT
	TTGACTTCATGACGTTATTTACGTAGAAT
	TTGACTTTGACAAAAAGTTTATATATAAT
	TTGAAAAATTATAGATTAGTGTTTATAAT
	TTGACAATAATATAAAAATATTATAATAT
	TTGAAAGTATTCTAAATGTTGATTATAAT
	TTGACTAAAAGGTTATAATATGTTATTAT
	TTTACTAAAAACTATGAAAATTTTACAAT
	TTGAAACTTTAAAAAAAATATAATATAAT
	TTTACAATATTGTTTTTATAGTATAAAAT
	TTTACAAAGTTTGATATAATAACTATAAT
	TTGACTAAAAGTATTTCTTATTTTAATAT
	TTTAATAAGCACGTACGGTTCGATAAAAT
	TTGACAAAAAAACTAAACAGTATGTAAAAT
	TTGACTTGTTAATACATCCATAGTATAAT

	TTGACATTTATCTCTATAGCTAATATAAT
	TTGACATTAGTGCCCAATTATAATATAAT
	TTGACTTACCTAATAAAAGTATATAATAT
	TTTAATATATATGAAAATAATAGTAAAAT
	TTGAATAATGGTTATTAAAAGTATATAAT
	TTGACTATATTAATTAAAAGTATTAAACT
	TAGACTTTATATATTTAATGTATTATAAT
	TTTACAAAATTTATAGAACAATATATAAT
	TTGACTTTGAAATAAGTATTTGATACTAT
	TTTAATTATTTCCAAGGAAATGTTATAAT
	TTGACAAAGATTATCAAATGATATAAAAT
	TAGACATAATGCAAGGATATGTATAAAAT
	TTGACAGAATGTTCGTAATGATATAAAAT
	TTGACAAATAATTTTTTTATTATATAAT
	TTTACTCTAAATATAATTAATTATATAAT
	TTGAAAAAAAGTATTTTTATAGGTATAAT
	TTTAATTATTTACACTTATAGGTTAAAAT
	TTGAAATTCAAATATATTTAATATACAAT
	TAGACAAATTTAAAAGCAGACTTTAAAAT
	TTGAATTTATCTTTTTTATGAAGTATAAT
	TTTACATAAAAGCATATATAATTTACAAT
	TATACTCCCTAAATAGTATATACTAAAAT
	TTGACATAGTGTATTTTTTATTGTATAAT
	TTGAAAAAATTTATAAATTATATATAAT
	TTGAATATATAAATTTAATAAAGTATAAT
	TTGACTTCCTATATTTATTGTTATATTAT
	TATACTGTGCAGTTTGTTAATTTTATAAT
	TTGACATAATTGCAAAAATAATGTATTAT
	TTGAAAAACATTTTTAATTACTATATAAT
	TTGAAAATAAGTTTTGTAAATGATATAAT
	TATACTCTGATATAATAAATTTATAAAAT
	TTGACAATGTTTGTAATATAACTTATTAT
	TTTAATTATTTACCATTTTTGCATAAAAT
	TTGACAGGCACACATTATAGTATTAAAAT
	TTTACAAGTATTTGAATTAGGTGTACAAT
	TTTAATTTTTACTTTTATTGTGATATAAT
	TTTACAAACATCTTTTTATATTTTACAAT
	TATACTATTAAATTTTAAAATTATATAAT
	TTTAATTATTTTTATATCATAGTTAAAAT
	TTGACTAATATAGGTCTATATATTAAAAT
	TTTACATTATAAATAAAATATTCTAAAAT
	TTTACACAATCGATATTTTTAGATATAAT
	TTTAATCTTTACTATATTAAGATTAAAAT
	TTTAATCTTAATATAGTAAAGATTAAAAT
	TTGACACACCGGGCAGATTGTATTATAAT
	TTGACATTGTACTTTCAAAATGATAAAAT
	TTGACAAAGCTTATGTAATATGATAATAT
	TTGACAATAAAAACCGATTTTACTATAAT
	TTTAATACTACAGTTATAATAATTAAAAT
	TTGAAAAAGTACTATCTAGTAAATAAAAT
	TTGACAAATTGCAAAAAAGCAATTATAAT
	TTGACAAATTGCAAAAAAGCAATTAAAAT
	TTGACAAAGAACAACAAATTTGATAAGAT
	TTGAAAAACATCATTATATCTGTTATAAT
	TTGACAGGATAAAGTACCTGTTTTATAAT
	TTGACATAGTATAGTTTGTGGGATATAAT
	TTGAAAATAATTTTTATATAAAATATAAT
	TTGAAAATACAGAAGGAAAAACATAAAAT
	TTTAATTCTTATATACTTGACTTTATAAT
	TTGAAAAAAAAGTATCTGTGGCATATAAT

	TTGACATATAAAAATATTAATTATAATAT<br>TTGACAAACATTCTTTGCTAAAGTAGAAT<br>TTTACTTTAATATTACAAATTGTTATAAT<br>TTGAATTTTCAATATATTAATGATAAAAT<br>TTTACTTTCAAATAACATACTTCTACAAT<br>TTGACATTATAAGACTACAAATATATAAT
C. butyricum DSM 10702 (NZ_CP040626.1)	**142 promoters,**<br>TTGACAGAAATTTATGTTGTATATATAAT<br>TTGACTAATTAAATTTTGTTATGTAAAAT<br>TATACTGCCACCACCTTTCACACTATAAT<br>TTGAAATTTTTTGAATTAAATGATATAAT<br>TTGACTTTTACTGTGTCATAAAATATAAT<br>TTGAAAATCCTACTTTTATTTTTTAAAAT<br>TTGACACATAAACAGAAATTCTGTATTAT<br>TTGACTGAAAAAATGTGTTTAAATAAATT<br>TATACTCCTTTTATATGAGACAATATAAT<br>TTTACAAAGAGGAAATATTGGTATATAAT<br>TGGACTAAGGAGCTTTATCTTTCTATAAT<br>TTGACACATGTCAAACCAAGTGATATACT<br>TTGACACATGTCAAACCAAGTGATATACT<br>TTGACACATGTCAAACCAAGTGATATACT<br>TTGACACATGTCAAACCAAGTGATATACT<br>TTGACATTTTATAATATTACATATAAAAT<br>TTTAATACTTGATGAAAAATATTTAAAAT<br>TTTACAAAAATTTATATTAGTGGTATAAT<br>TATACTGGGCTTGGTGGAAATAATACAAT<br>TTGACTAGGATAATATTTAAGAGTAATAT<br>TTGACATTGGTAAAATTTAATAATATACT<br>TATACTGAATAATAGTGATAATATAAAAT<br>TTGACAATTGGGGTTAATTATAATAGAAT<br>TTGACTACGATTTCATAAAGTAGTAATAT<br>TTGAAAGCCATAATGATATAACATATAAT<br>TTGAATTTCTATAGAGTAAAATTTATAAT<br>TTGAATTTACTCTTTATACTCAATACAAT<br>TTGACAATTCTGTTATGGAAATATATTAT<br>TTTAAATTAAATAAGCCAATACCTAAAAT<br>TTTACATCTTCCCATTATAGTTTTAAAAT<br>TTGACAAAAAATAAACAATACACTAAAAT<br>TTTAAACAAGCAGGAGAAGAATTTAAAAT<br>TTTAAATATACATAGGATAAATTTAAAAT<br>TTGAATTTATGTATTATTAAAGATAAAAT<br>TTGACATGCAGGATTTTATTTTGTATAAT<br>TTTACTGGATCATAAGATTCTTCTAAAAT<br>TATACTTAATGTAGTTATCAAAATAAAAT<br>TTTAATTCCTCTGATTCCACCTATAAAAT<br>TTGACATAAATAGAGAAAAAGTATAAACT<br>TTTAATGTCTAGAAACAAGTTTATAAAAT<br>TTGACAGTCATTTACATTAAATATATAAT<br>TTGACACAAGTCAAACCAAGTGATATACT<br>TATACTCTGTCATACCCATCTATTAAAAT<br>TTGACATGAAATGCTTCGAGTGATATACT<br>TTGACAGTATACATAATAGTCTGTATAAT<br>TTGACATATACGGATTAAGTTTATATAAT<br>TTTAATTAAAGAAGACTTAAAGCTAAAAT<br>TTGAAAAATATTTAATTTAATTATATAAT<br>TTGACTTTCGGTAAAATTTATTATAATAT<br>TTGACATATTAGTCATATCTCGATATAAT<br>TTGACATGTCCTAAAGTTCACTATATAAT<br>TTGACTTTATATTACATTCTTGGTAATAT<br>TTTAATGCTTTCTTATGTTTATTTAAAAT

	TTGACTTACTGAATAAGGAAAAGTACAAT
	TTTACAAAAATAAAAACTTATAGTATAAT
	TTGACTGGAATTGTAGCTATACCTACGAT
	TTGAAAAAAATATAAATAATATATATAAT
	TTTAATGGATGTTACTACTGCTATAAAAT
	TTTACATCAGTTTTAATAAATTTTAAAAT
	TTTACGCCACCTACTTCTGCAAGTATAAT
	TTTAATTTTTTATAAATAAATATTAAAAT
	TTTAATATTTATTTATAAAAAATTAAAAT
	TTGAATAATTATAAAAATGTATGTATAAT
	TTTACTCATTTTGTTTATTAGTTTATAAT
	TTTACATATTATACTTATATATCTACAAT
	TTGAAACATTGTAAAAGTTAGGTTATAAT
	TTTAATTTGGATATATGTATAAATACAAT
	TTTACATGAAATAAAATTGATGTTACAAT
	TATACTAATACTATATTATTTGATATAAT
	TTTAATACTCCTGTGATAATTTTTAAAAT
	TATACTTTAATTATTAGTATACCTATAAT
	TTTAATAATGGGGCATATAGTTATAAAAT
	TTTACTATATCATTTTCACAGAATATAAT
	TTGAAAAATCACAAAATTTGCCATATAAT
	TTTAATCATTTCTGGGATATTTTTATAAT
	TTTACTAAATTGGGTTCATTTTTTAAAAT
	TTGACTTTTGGAATAAATGGGAATATAAT
	TTTACAGAAGATAATTTCATAGATAGAAT
	TGGACTTCTTACTATTTTTAGGGTATAAT
	TTGACAATATATTTTATAAAATGTAAACT
	TTTAAAGAACATAACATTAATATTAAAAT
	TTTAAAATATAAAAAGAATTCAGTAAAAT
	TTTAATGCTTTCAATTTTCACACTATAAT
	TTGACTTAACTTTTGTCAAACCTTAAACT
	TTGACCAAATTTGGGGGTATTTTTATAAT
	TTTAATATCTATTTCTTCCGTAGTAAAAT
	TTTACTAAGACAGCTGCCTGTGGTACAAT
	TTTAATTTATAATAGTAAGTAAGTATAAT
	TTGAATAATAATTATTATTGGTATAAAAT
	TATACTATTTATACTTACTAGGATATAAT
	TTGAAATCTCTTTTTTGTATTAATACAAT
	TTTACAACGGATAATTATTATTATATAAT
	TTTACTTATTAAATAGTTTTATGTAGAAT
	TTTACTTACGACTATTATACTTTTAAAAT
	TTGACTTTTTTTACGTAGATATATACAAT
	TTGACTTGGGTGGCCATTAAAATTAATAT
	TTGAAAAACGTTTTTGGTAAAGGTAAAAT
	TTGACATAAAAAAATGTTTGAACTATAAT
	TTTACATGAAGAGTGGTTAAAAATATAAT
	TTTAAAAAACTTAACCTTTTAATTATAAT
	TTGACTATGAGGAAAATAGCAATTATAAT
	TTTACAGACCATGTAGATTACGATATAAT
	TTGACAATAGCTTTTCTTAGTATTATACT
	TTTACTAAAATAAATCTGTTTTTTATAAT
	TTTAATTCTTATGTTATTCTACTTATAAT
	TTGAAAAGAAGACAAACTACTTTTATAAT
	TTTACGTGGAAATATGTCAGAAATAAAAT
	TTGACTCTACAAGTGAAGAAATGTATGAT
	TTGAATAGATTATGAACGAGTATTATAAT
	TTTAATTTATCAATTATTTGTATTATAAT
	TTGAATTAAATTAAATAAAATAGTAGAAT
	TTTACATCAACAAGTATAACCCCTATAAT
	TTTAATAGTTTTCAAGAAAAACATAAAAT

	TTTAAAAGTGATATTATTTCATCTAAAAT
	TTTAAACCATGGAAAGGATGATTTATAAT
	TTTAATAAATAAAAAAATATTCTTATAAT
	TTTAAATTATGGGTTATAGATTTTAAAAT
	TTTACACTTAGAAGGTGAAGTGATACAAT
	TTGACTTCTTGCTGGTACATGAGCATAAT
	TTGACATTGTACTATATTTATTGTAAAAT
	TTTAATTTATTTGTACTAATTTATAAAAT
	TTTACGTCTTTTACAATATCAGATAAAAT
	TTGAATATAGTTATTTTTAATCATATAAT
	TTGAAAACCTTTTAAAAATGCTTTATAAT
	TTGACTTTTTACTAAAATAAATATAAAAT
	TTGAATATGTCCAGGTACTTCAATATAAT
	TTTAATGATTTTTCTTATTATGTTATAAT
	TTTACTAATCTTTCATGATAATTTAAAAT
	TTGACAATAAATTTTTATAGTAGTAATAT
	TTGACATACATTAAAATGGTTGTTATTAT
	TTGAATTTAAAGAAAAAAATATATATAAT
	TTGAAAATTTACAAGAAAAGAATTATAAT
	TATACTAAAGCAGCATACTGCTATATAAT
	TTGACATAGTATGATTAATAATATATAAT
	TTGAAAATATATACCCATAGGGGTATAAT
	TTGAAATTTTATTTATGTTATAATAAAAT
	TTGAATTCAATATATTCAATCATTACAAT
	TTGACAACTTTCATATAATTTTATACTAT
	TTGACACTGACAAAATCATAACGTAAAAT
	TTTAATTTTAATTTACTTGAAACTAAAAT
	TTTAAATATAAAAATGAAGATTATATAAT
	TTGACTTGAAATAAATAGCAATATATAAT

Table S4. Sequences of *sacB* expression cassettes. Promoters-5’ UTR are underlined; start codons are highlighted in bold; the insertion sequence is highlighted in red.

***sacB* expression cassettes**	**5’ - 3’ Sequences**
P*sacB*-AUG*sacB*	CACATATACCTGCCGTTCACTATTATTTAGTGAAATGAGATATTATGAT<br>ATTTTCTGAATTGTGATTAAAAAGGCAACTTTATGCCCATGCAACAGAA<br>ACTATAAAAAATACAGAGAATGAAAAGAAACAGATAGATTTTTTAGTT<br>CTTTAGGCCCGTAGTCTGCAAATCCTTTTATGATTTTCTATCAAACAAAA<br>GAGGAAAATAGACCAGTTGCAATCCAAACGAGAGTCTAATAGAATGAG<br>GTCGAAAAGTAAATCGCGCGGGTTTGTTACTGATAAAGCAGGCAAGAC<br>CTAAAATGTGTAAAGGGCAAAGTGTATACTTTGGCGTCACCCCTTACAT<br>ATTTTAGGTCTTTTTTTATTGTGCGTAACTAACTTGCCATCTTCAAACAG<br>GAGGGCTGGAAGAAGCAGACCGCTAACACAGTACATAAAAAAGGAGA<br>CATGAACG**ATG**AACATCAAAAAGTTTGCAAAACAAGCAACAGTATTAA<br>CCTTTACTACCGCACTGCTGGCAGGAGGCGCAACTCAAGCGTTTGCGAA<br>AGAAACGAACCAAAAGCCATATAAGGAAACATACGGCATTTCCCATAT<br>TACACGCCATGATATGCTGCAAATCCCTGAACAGCAAAAAAATGAAAA<br>ATATCAAGTTCCTGAATTCGATTCGTCCACAATTAAAAATATCTCTTCTG<br>CAAAAGGCCTGGACGTTTGGGACAGCTGGCCATTACAAAACGCTGACG<br>GCACTGTCGCAAACTATCACGGCTACCACATCGTCTTTGCATTAGCCGG<br>AGATCCTAAAAATGCGGATGACACATCGATTTACATGTTCTATCAAAAA<br>GTCGGCGAAACTTCTATTGACAGCTGGAAAAACGCTGGCCGCGTCTTTA<br>AAGACAGCGACAAATTCGATGCAAATGATTCTATCCTAAAAGACCAAA<br>CACAAGAATGGTCAGGTTCAGCCACATTTACATCTGACGGAAAAATCCG<br>TTTATTCTACACTGATTTCTCCGGTAAACATTACGGCAAACAAACACTG<br>ACAACTGCACAAGTTAACGTATCAGCATCAGACAGCTCTTTGAACATCA<br>ACGGTGTAGAGGATTATAAATCAATCTTTGACGGTGACGGAAAAACGT<br>ATCAAAATGTACAGCAGTTCATCGATGAAGGCAACTACAGCTCAGGCG<br>ACAACCATACGCTGAGAGATCCTCACTACGTAGAAGATAAAGGCCACA<br>AATACTTAGTATTTGAAGCAAACACTGGAACTGAAGATGGCTACCAAG<br>GCGAAGAATCTTTATTTAACAAAGCATACTATGGCAAAAGCACATCATT<br>CTTCCGTCAAGAAAGTCAAAAACTTCTGCAAAGCGATAAAAAACGCAC<br>GGCTGAGTTAGCAAACGGCGCTCTCGGTATGATTGAGCTAAACGATGAT<br>TACACACTGAAAAAAGTGATGAAACCGCTGATTGCATCTAACACAGTA<br>ACAGATGAAATTGAACGCGCGAACGTCTTTAAAATGAACGGCAAATGG<br>TACCTGTTCACTGACTCCCGCGGATCAAAAATGACGATTGACGGCATTA<br>CGTCTAACGATATTTACATGCTTGGTTATGTTTCTAATTCTTTAACTGGC<br>CCATACAAGCCGCTGAACAAAACTGGCCTTGTGTTAAAAATGGATCTTG<br>ATCCTAACGATGTAACCTTTACTTACTCACACTTCGCTGTACCTCAAGCG<br>AAAGGAAACAATGTCGTGATTACAAGCTATATGACAAACAGAGGATTC<br>TACGCAGACAAACAATCAACGTTTGCGCCAAGCTTCCTGCTGAACATCA<br>AAGGCAAGAAAACATCTGTTGTCAAAGACAGCATCCTTGAACAAGGAC<br>AATTAACAGTTAACAAATAA
P*thl*_fU-AUG*sacB*-insert sequence	TTTTTAACAAAATATATTGATAAAAATAATAATAGTGGGTATAATTAAC<br>GTATAAAGTTGTGTAATTTTTAAGGAGGTGTGTTACAT**ATG**AACATCAA<br>AAAGTTTGCAAAACAAGCAACAGTATTAACCTTTACTACCGCACTGCTG<br>GCAGGAGGCGCAACTCAAGCGCTGATGAATCCCCTAATGATTTTTATCA<br>AAATCATTAAGTTAAGGTAGATACACATCTTGTCATATGATCAAATGGT<br>TTCGCCAAAAATCAATAATCAGACAACAAAATGTGCGAACTCGATATTT<br>TACACGACTCTCTTTACCAATTCTGCCCCGAATTACACTTAAAACGACTC<br>AACAGCTTAACGTTGGCTTGCCACGCCTTACTTGACTGTAAAACTCTCA<br>CTCTTACCGAACTTGGCCGTAACCTGCCAACCAAAGCGAGAACAAAAC<br>ATAACATCAAACGAATCGACCGATTGTTAGGTAATCGTCACCTCCACAA<br>AGAGCGACTCGCTGTATACCGTTGGCATGCTAGCTTTATCTGTTCGGGC<br>AATACGATGCCCATTGTACTTGTTGACTGGTCTGATATCCGTGAGCAAA<br>AACGGCTTATGGTATTGCGAGCTTCAGTCGCACTACACGGTCGTTCTGT<br>TACTCTTTATGAGAAAGCGTTCCCGCTTTCAGAGCAATGTTCAAAGAAA<br>GCTCATGACCAATTTCTAGCCGACCTTGCGAGCATTCTACCGAGTAACA<br>CCACACCGCTCATTGTCAGTGATGCTGGCTTTAAAGTGCCATGGTATAA<br>ATCCGTTGAGAAGCTGGGTTGGTACTGGTTAAGTCGAGTAAGAGGAAA<br>AGTACAATATGCAGACCTAGGAGCGGAAAACTGGAAACCTATCAGCAA

	CTTACATGATATGTCATCTAGTCACTCAAAGACTTTAGGCTATAAGAGG<br>CTGACTAAAAGCAATCCAATCTCATGCCAAATTCTATTGTATAAATCTC<br>GCTCTAAAGGCCGAAAAAATCAGCGCTCGACACGGACTCATTGTCACC<br>ACCCGTCACCTAAAATCTACTCAGCGTCGGCAAAGGAACCAGGGATTCT<br>AGCAACTAACTTACCTGTTGAAATTCGAACACCCAAACAACTTGTTAAT<br>ATCTATTCGAAGCAAAGGCAAATTGAAGAAACCTTCCGAAACTTGAAA<br>AGTCCTGCCTACGGACTAGGCCTACCCCATNACCGAACGAGCAATCCAA<br>AACGTTTTGAATACAGGNCGCCAAACCCCCTGGGGCTTCAACTAAATGG<br>GNCTTTGGGGNGGTNNNTGGNCAAAAAAAGGGTGGAAAAACCTTTCG<br>GGACAAACCTCAAAAAAAAAAAGCTATCTNCCAAATTCTTTANNGGGAA<br>ATTTCC
P*thl*_fU-UUG*sacB*	TTTTTAACAAAATATATTGATAAAAATAATAATAGTGGGTATAATTAAC<br>GTATAAAGTTGTGTAATTTTTAAGGAGGTGTGTTACAT**TTG**AACATCAA<br>AAAGTTTGCAAAACAAGCAACAGTATTAACCTTTACTACCGCACTGCTG<br>GCAGGAGGCGCAACTCAAGCGTTTGCGAAAGAAACGAACCAAAAGCCA<br>TATAAGGAAACATACGGCATTTCCCATATTACACGCCATGATATGCTGC<br>AAATCCCTGAACAGCAAAAAAATGAAAAATATCAAGTTCCTGAATTCG<br>ATTCGTCCACAATTAAAAATATCTCTTCTGCAAAAGGCCTGGACGTTTG<br>GGACAGCTGGCCATTACAAAACGCTGACGGCACTGTCGCAAACTATCA<br>CGGCTACCACATCGTCTTTGCATTAGCCGGAGATCCTAAAAATGCGGAT<br>GACACATCGATTTACATGTTCTATCAAAAAGTCGGCGAAACTTCTATTG<br>ACAGCTGGAAAAACGCTGGCCGCGTCTTTAAAGACAGCGACAAATTCG<br>ATGCAAATGATTCTATCCTAAAAGACCAAACACAAGAATGGTCAGGTTC<br>AGCCACATTTACATCTGACGGAAAAATCCGTTTATTCTACACTGATTTCT<br>CCGGTAAACATTACGGCAAACAAACACTGACAACTGCACAAGTTAACG<br>TATCAGCATCAGACAGCTCTTTGAACATCAACGGTGTAGAGGATTATAA<br>ATCAATCTTTGACGGTGACGGAAAAACGTATCAAAATGTACAGCAGTTC<br>ATCGATGAAGGCAACTACAGCTCAGGCGACAACCATACGCTGAGAGAT<br>CCTCACTACGTAGAAGATAAAGGCCACAAATACTTAGTATTTGAAGCAA<br>ACACTGGAACTGAAGATGGCTACCAAGGCGAAGAATCTTTATTTAACA<br>AAGCATACTATGGCAAAAGCACATCATTCTTCCGTCAAGAAAGTCAAA<br>AACTTCTGCAAAGCGATAAAAAACGCACGGCTGAGTTAGCAAACGGCG<br>CTCTCGGTATGATTGAGCTAAACGATGATTACACACTGAAAAAAGTGAT<br>GAAACCGCTGATTGCATCTAACACAGTAACAGATGAAATTGAACGCGC<br>GAACGTCTTTAAAATGAACGGCAAATGGTACCTGTTCACTGACTCCCGC<br>GGATCAAAAATGACGATTGACGGCATTACGTCTAACGATATTTACATGC<br>TTGGTTATGTTTCTAATTCTTTAACTGGCCCATACAAGCCGCTGAACAAA<br>ACTGGCCTTGTGTTAAAAATGGATCTTGATCCTAACGATGTAACCTTTA<br>CTTACTCACACTTCGCTGTACCTCAAGCGAAAGGAAACAATGTCGTGAT<br>TACAAGCTATATGACAAACAGAGGATTCTACGCAGACAAACAATCAAC<br>GTTTGCGCCAAGCTTCCTGCTGAACATCAAAGGCAAGAAAACATCTGTT<br>GTCAAAGACAGCATCCTTGAACAAGGACAATTAACAGTTAACAAATAA
